# Biomarker-focused multi-drug combination therapy and repurposing trial in *mdx* mice

**DOI:** 10.1371/journal.pone.0246507

**Published:** 2021-02-22

**Authors:** Michael Ziemba, Molly Barkhouse, Kitipong Uaesoontrachoon, Mamta Giri, Yetrib Hathout, Utkarsh J. Dang, Heather Gordish-Dressman, Kanneboyina Nagaraju, Eric P. Hoffman

**Affiliations:** 1 Department of Biomedical Engineering, Binghamton University–State University of New York, Binghamton, NY, United States of America; 2 AGADA Biosciences, Halifax, Nova Scotia, Canada; 3 Department of Genetics and Genomic Sciences, Mount Sinai Hospital, New York, NY, United States of America; 4 Department of Pharmaceutical Sciences, School of Pharmacy and Pharmaceutical Sciences, Binghamton University–State University of New York, Binghamton, NY, United States of America; 5 Department of Health Outcomes and Administrative Sciences, School of Pharmacy and Pharmaceutical Sciences, Binghamton University–State University of New York, Binghamton, NY, United States of America; 6 Center for Translational Sciences, Children’s National Medical Center, Washington, DC, United States of America; University of Minnesota Medical School, UNITED STATES

## Abstract

Duchenne muscular dystrophy is initiated by dystrophin deficiency, but downstream pathophysiological pathways such as membrane instability, NFĸB activation, mitochondrial dysfunction, and induction of TGFβ fibrosis pathways are thought to drive the disability. Dystrophin replacement strategies are hopeful for addressing upstream dystrophin deficiency; however, all methods to date use semi-functional dystrophin proteins that are likely to trigger downstream pathways. Thus, combination therapies that can target multiple downstream pathways are important in treating DMD, even for dystrophin-replacement strategies. We sought to define blood pharmacodynamic biomarkers of drug response in the *mdx* mouse model of Duchenne muscular dystrophy using a series of repurposed drugs. Four-week-old *mdx* mice were treated for four weeks with four different drugs singly and in combination: vehicle, prednisolone, vamorolone, rituximab, β-aminoisobutyric acid (BAIBA) (11 treatment groups; n = 6/group). Blood was collected via cardiac puncture at study termination, and proteomic profiling was carried out using SOMAscan aptamer panels (1,310 proteins assayed). Prednisolone was tested alone and in combination with other drugs. It was found to have a good concordance of prednisolone-responsive biomarkers (56 increased by prednisolone, 39 decreased) focused on NFκB and TGFβ cascades. Vamorolone shared 45 (80%) of increased biomarkers and 13 (33%) of decreased biomarkers with prednisolone. Comparison of published human corticosteroid-responsive biomarkers to our *mdx* data showed 14% (3/22) concordance between mouse and human. Rituximab showed fewer drug-associated biomarkers, with the most significant being human IgG. On the other hand, BAIBA treatment (high and low dose) showed a drug-associated increase in 40 serum proteins and decreased 5 serum proteins. Our results suggest that a biomarker approach could be employed for assessing drug combinations in both mouse and human studies.

## Introduction

Duchenne muscular dystrophy (DMD) is an X-linked, progressive, degenerative muscle disease caused by loss of dystrophin in muscle tissues. DMD patients show progressive muscle degeneration, loss of gross motor skills, and early death. DMD affects approximately one in 5000 live male births and causes loss of mobility by the early teens and death by their early twenties unless ventilated. Dystrophin is present in all skeletal and cardiac muscle from early fetal life and absent in all DMD tissues from fetal life onward [1]. However, different muscle types show different age-related pathological responses to dystrophin, with some skeletal muscle types such as sartorius and heart showing little or no evidence of dystrophic changes. In contrast, other skeletal muscles show progression to fibrofatty replacement and loss of function [2, 3]. Some of the variable pathological responses of different muscle groups have been attributed to TGFβ pathways and asynchronous regeneration [4]. It is clear that dystrophin deficiency initiates a series of pathological events over many years in a subset of DMD patient muscles, and this drives disability and early death, but dystrophin deficiency itself may not be ‘sufficient’ for disability.

The current recommended treatment for DMD is the use of corticosteroids that slow the progression of the disease and delay the loss of ambulation. Although drugs have been approved for exon skipping (Eteplirsen; FDA), specific corticosteroids (Emflaza [deflazacort]; FDA), and stop codon read-throughs (Translarna; EMA), neither Eteplirsen nor Translarna have demonstrated compelling evidence of clinical efficacy, and deflazacort shows a more severe safety profile than prednisone [5].

Long-term use of corticosteroids results in severe and well documented side effects for the patient. The side effects of corticosteroids negatively impact life quality by increasing body weight, stunting growth, delaying puberty, and causing mood disturbances. In addition, corticosteroids negatively alter bone remodeling leading to a higher prevalence of osteopenia, scoliosis, and fractures in treated patients. Alternative drug therapies with similar anti-inflammatory effects as corticosteroids, but with fewer and less severe side effects than prednisone, are being proposed, such as vamorolone, a first-in-class steroidal drug currently in first-in-patient studies in DMD [6–8].

Due to the various pathophysiological pathways such as membrane instability, NFĸB activation, mitochondrial dysfunction, and induction of TGFβ fibrosis, models where multiple drugs are used to target different biochemical pathways in DMD have been suggested. Successful treatment of DMD could likely be a ‘combination therapy’ approach. While it is tempting to suggest that dystrophin replacement alone would abrogate the need for combination therapies targeting downstream pathways, this viewpoint is likely naïve as all dystrophin replacement therapies involve semi-functional (Becker-like) dystrophin proteins that initiate similar pathological cascades as DMD.

Combination therapies in DMD could involve drugs aimed at targeting myofiber membrane stability (vamorolone [9]) or inflammation (inhibition of NFĸB [prednisone, deflazacort, vamorolone], or B lymphocytes [rituximab]). Dystrophin deficient muscle also shows defects in cell metabolism, particularly oxidative metabolism, and there are efforts to use drugs to bolster mitochondrial function [10, 11]. One possibility to provide a salvage pathway for oxidative metabolism [12, 13] could be β-aminoisobutyric acid (BAIBA), as it increases brown fat function [12].

A difficulty with the development of combination therapies is the multiple pharmacological variables that become challenging to control, such as dose-optimization of each drug, drug-drug-interactions (DDI), and monitoring aspects of both efficacy and safety attributable to each drug singly and in combination. Blood protein biomarkers provide one attractive alternative for use as pharmacodynamic tools in the development of combination therapies [14]. Blood protein biomarkers have been used previously to monitor disease progression in DMD, [15] assess corticosteroid-response in DMD [16] and concordance with corticosteroid-response in other inflammatory states, [17] and to assess anti-inflammatory effects of a novel drug, vamorolone, in DMD [6]. In each of the aforementioned studies, SOMAscan aptamer panels were used to test 1,310 proteins simultaneously. The SOMAmer aptamers used in the SOMAscan assay each have a very high affinity for a particular human protein, coupled with a specific unique nucleotide sequence used as an address for detecting hybridization of DNA probes. The SOMAmers are then hybridized to custom DNA microarrays, which allows the amount of protein to be measured in fluorescent intensity. At the time of this study, the SOMAscan assay measured 1,310 proteins using SOMAmers developed and included in their assay.

This study utilized the SOMAscan aptamer platform to assess pharmacodynamic biomarkers in the *mdx* mouse treated with four drugs singly and in combination (prednisolone, vamorolone, rituximab, and β-aminoisobutyric acid). The muscle microenvironment of dystrophin-deficient muscle is complex, with multiple pathological pathways operating simultaneously [18]. It is highly unlikely that blocking or enhancing one pathway will give a significant therapeutic benefit. After carefully evaluating biological and pathological pathways, we proposed that maximal benefit could occur if we simultaneously block immune-inflammatory pathways along with enhancing metabolic pathways. To previously enhance an easy transition to clinical trials in DMD children, we have selected previously tested drugs in humans. Prednisolone is the active metabolite of prednisone and is a commonly utilized anti-inflammatory corticosteroid. Although commonly utilized, corticosteroids prednisone, prednisolone, and deflazacort cause many side effects that detract from DMD patient quality of life [19]. Prednisolone enters the cell by passive diffusion through plasma membranes, binds corticosteroid receptors (GR), dimerizes, then binds positive gene promoter elements on genomic DNA (corticosteroid-response elements; GREs; termed transactivation), and distinct negative NFκB binding promoter element (termed transrepression) [20]. Vamorolone is a first-in-class small molecule steroidal drug that has multiple activities of potential benefit to DMD, including improvement of plasma membrane repair, [21] NFĸB inhibition via transrepression [21], potent antagonism of the mineralocorticoid receptor [22], and inhibition of microRNAs that reduce dystrophin mRNA translation [23]. Rituximab is a monoclonal antibody with specificity for CD20 (CD20 positive B cells are often noted in dystrophin-deficient muscle), a B lymphocyte surface marker, and treatment leads to B cell depletion [24]. In addition, Rituximab is known to bind to alternative targets such as sphingomyelinase-like phosphodiesterase 3b (SMPDL3B) that are also expressed in many cell types, including skeletal muscle [25]. BAIBA is a small molecule non-protein amino acid originating from the catabolism of thymine and valine created by muscles during exercise that prepares fat for metabolism back into energy and increases insulin sensitivity [12]. BAIBA also activates AMPK (AMP-activated protein kinase), which is also an inhibitor of the NFκB inflammatory pathway [13].

## Materials and methods

### Animals

Four-week-old male, C57BL/10ScSn-Dmdmdx/J (BL-10-mdx) with a nonsense mutation in exon 23, *mdx* mice were used for this study. All animals (stock number: 001801) were bred and purchased from Jackson Laboratory (Bar Harbor, Maine, USA). Mice were weighed on arrival and acclimate for one week before assignment to dose groups and drug treatment. *mdx* mice were sorted into 11 groups by bodyweight after the acclimation period. The average weight of each group was within 5% of each of the other groups. Two cages were randomly assigned to each treatment group. Mice were housed 3 to a cage and identified by ear tags.

The Dalhousie University Animal Committee approved the animal work performed in this study following the Canadian Council on Animal Care. During the study, 13-hour light/11-hour dark cycles were maintained (06:30 AM-7:30 PM light, 7:30 PM-6:30 AM dark). All testing and drug treatment were performed during the light cycle phase. The room temperature was maintained between 20°C and 23°C. Food and water were available ad libitum for the duration of the study. Animals were provided with environmental enrichment and monitored daily for body weight changes, behavior, food intake, and respiratory system. *mdx* mice in this study were not subjected to procedures that cause excessive pain and suffering. To reduce pain and suffering, euthanasia was performed with a ketamine (80mg/kg)/xylazine (10mg/kg) cocktail, followed by cervical dislocation for all animals.

The *mdx* vehicle group was injected once with a saline solution and then were treated daily with oral cherry syrup. Mice being treated with prednisolone or vamorolone received treatment daily via cherry syrup at approximately the same time every day. Mice being treated with rituximab received one injection of 200μl via subcutaneous injection at the beginning of the treatment period. Groups treated with β-aminoisobutyric acid were treated by diluting the drug in drinking water. The BAIBA dose was adjusted based on the three mice’s water intake and average body weight in each cage to achieve the indicated mg/kg/day dose.

After four weeks of treatment, blood samples were collected by cardiac puncture under anesthesia, and the mice were euthanized by cervical dislocation. Blood was collected, allowed to clot, and then processed by centrifuging for 10 minutes at 10,000 rpm at 4°C to retain serum. Aliquots of serum were collected and stored at -80°C before being sent to SOMAlogic for SOMAscan profiling of proteins using aptamer panels.

### Ingenuity pathway analysis

Ingenuity Pathway Analysis (IPA) utilizes a large database of biological findings (published research) to determine causal networks involved with, in this case, proteomics data. When data is submitted to IPA, their algorithm searches the database for the proteins of interest and the interactions between the proteins submitted and any other proteins, genes, or other relevant material. The software then uses probabilities to determine the most probable networks associated with the data submitted. Since these results are based on previously studied association data, the networks will show all proteins in the network (whether they are significant or not) and their interaction via network edges or lines that indicate the interaction. If applicable, the direction of the interaction. The protein symbols and edges also serve as links to the supporting documentation for the results.

### Statistical analysis

The SOMAscan (SomaLogic, Boulder, Colorado) assay contains 1,310 results per sample, reported in Relative Fluorescent Units. Two data sets are provided by SOMAlogic, one with hybridization control normalization and median signal normalization and one set with hybridization control normalization only. The SOMAscan quality statement states that "when comparing the median normalization scale factors across the supplied groupings, there is the possibility that median normalization is removing something significant other than nuisance differences in overall protein concentration [26].

Shapiro-Wilk tests for normal distribution were run on all data using R statistical programming language (R Foundation for Statistical Computing, Vienna, Austria) at an alpha of p<0.05. A large number of result groups failed the Shapiro-Wilk test. The data was then subjected to a log base 10 transformation, and the Shapiro-Wilk test was re-run. Many result groups still failed the Shapiro-Wilk test; therefore, the nonparametric Mann-Whitney test was used with the original, non-transformed data to determine differential protein expression between groups.

## Results

### Proteomic profiling of drug-treated *mdx* mice

Four drugs (prednisolone, vamorolone, rituximab, and BAIBA) were used singly and in combination to treat *mdx* mice for 4 weeks (6 mice/groups) (**Table 1**Serum was collected after the treatment period, and proteomic profiling was performed using SOMAscan aptamers to detect serum proteins. Non-parametric Mann-Whitney tests were used to analyze the median-normalized data to determine differential proteins between groups.

**Table 1:** Drug treatment regimens used in dystrophin-deficient mdx mice.

Group	Strain	N	Treatment	Dosage
				(mg/kg/day)
**1**	*mdx*	6	Vehicle (saline then cherry syrup)	-
**2**	*mdx*	6	Prednisolone	5 mg/kg/day
**3**	*mdx*	6	Rituximab	1 mg/kg
				(1 injection)
**4**	*mdx*	6	β-aminoisobutyric acid	100 mg/kg/day
**5**	*mdx*	6	β-aminoisobutyric acid	500 mg/kg/day
**6**	*mdx*	6	Rituximab + Prednisolone	1 mg/kg +
				5 mg/kg/day
**7**	*mdx*	6	β-aminoisobutyric acid (low dose) + Prednisolone	100 mg/kg/day + 5 mg/kg/day
**8**	*mdx*	6	β-aminoisobutyric acid (high dose) + Prednisolone	500 mg/kg/day + 5 mg/kg/day
**9**	*mdx*	6	β-aminoisobutyric acid (low dose) + Rituximab + Prednisolone	100 mg/kg/day + 1 mg/kg +
				5 mg/kg/day
**10**	*mdx*	6	Vamorolone	30 mg/kg/day
**11**	*mdx*	6	Vamorolone + β-aminoisobutyric acid (low dose) + Rituximab	30 mg/kg/day + 100 mg/kg/day +
				1 mg/kg

The average weight of each treatment group was calculated at the start of the study and the end of the study. The percent change in body weight during the 4- week treatment period was calculated. The analysis of body weight over the study’s length between the mdx vehicle and mdx treatment groups showed that the relationship between body weight and time was shown to be linear for all mdx mice groups. Note the mdx vehicle mice are the reference group. Several treatment groups showed a significant difference from vehicle-treated mice when assessing overall time points (**Table 2**). All of the treatment groups containing prednisolone and group 11 containing vamorolone showed a significant decrease in body weight from vehicle-treated mice over all time points (i.e., a negative β coefficient). Mice treated with β-aminoisobutyric acid (high dose) showed a significant increase in body weight from vehicle-treated mice over all time points (i.e., a positive β coefficient). In addition, there was a significant effect of the time for all mice combined; mean body weight increased on average 1.46 g per week (p<0.001). The interaction between time and treatment group(s) was not tested here.

**Table 2:** Analysis of body weight over time between MDX vehicle and MDX treatment groups.

	Comparison groups	Treatment group term		Time term	
Group #		β	P-value	β	p-value
1	MDX vehicle (referent group)	---		1.46	<0.001
2	Prednisolone	-2.21	0.020		
3	Rituximab	0.74	0.44		
4	β-aminoisobutyric acid (low dose)	1.93	0.043		
5	β-aminoisobutyric acid (high dose)	0.34	0.72		
6	Rituximab + prednisolone	-2.56	0.007		
7	β-aminoisobutyric acid (low dose) + prednisolone	-2.56	0.007		
8	β-aminoisobutyric acid (high dose) + prednisolone	-3.47	<0.001		
9	β-aminoisobutyric acid (low dose)+ prednisolone + Rituximab	-3.33	<0.001		
10	Vamorolone	-1.62	0.09		
11	Vamorolone + β-aminoisobutyric acid (low dose)+ Rituximab	-2.36	0.013		

### Corticosteroid-responsive serum biomarkers

Prednisolone was administered to 5 different groups of mice, either alone (Group 2) or in combination with other drugs (Groups 6, 7, 8, 9) (**Table 1**). The number of mice in each group (n = 6) was insufficient to adjust for multiple testing of 1,310 protein tests statistically. Thus, we employed a ’discovery’ data set (vehicle [Group 1] vs. prednisolone [Group 2]), with an unadjusted p-value threshold of p<0.01. Any serum proteins significantly changed by prednisolone alone were then tested as ’candidates’ in the other groups that included prednisolone treatment (Groups 6, 7, 8, 9). These were not independent validations, as the comparisons to the later groups were against the same vehicle control (Group 1).

Comparisons of the vehicle (Group 1) vs. prednisolone alone (Group 2) showed 13 proteins with p<0.01. Per our statistical analysis plan, these results were then used in pairwise comparisons against Groups 6, 7, 8, and 9, where each biomarker needed to show p<0.05 in at least three of the four validation groups to be retained for comparison to known corticosteroid-responsive biomarkers, to vamorolone.

This analysis showed 73 proteins to be up-regulated by prednisolone, and 64 proteins down-regulated (p<0.01; Group 2 vs. Group 1 only) (**Table 3**; **S1 Table in S1 File**). 69% of these prednisone-responsive proteins validated in 3 or 4 prednisone-containing multi-drug groups (n = 56 upregulated, n = 39 downregulated; p<0.05 each comparison); the direction of change was consistent between-group comparisons (**S1 Table in S1 File**). The fold changes for these 95 proteins were plotted for the prednisolone validation groups (G6, G7, G8, and G9). The plots are shown in **S1A Fig in S1 File**. Except for some potential outliers, these plots are unremarkable. A Venn diagram showing these results graphically is in **S2A Fig in S1 File**. All these proteins are listed in the S2 File.

**Table 3:** Serum proteomics comparisons of drug-treated vs. non-treated controls (Group 1) for prednisolone-treated and vamorolone-treated mdx mice.

	Group 2 prednisolone vs. Group 1 control	Group 6 Prednisolone + Rituximab vs. Group 1 control	Group 7 Prednisolone + βAIBA (low dose) vs. Group 1 control	Group 8 Prednisolone + βAIBA (high dose) vs. Group 1 control	Group 9 Prednisolone + βAIBA (low dose) + Rituximab vs. Group 1 control	Prednisolone concordant (4 of 5 groups)	Group 10 Vamorolone vs. Group 1 control	Group 11 Vamorolone + βAIBA (low dose) + Rituximab vs. Group1 control	Prednisolone and vamorolone concordant
	P<0.01	P<0.05	p<0.05	p<0.05	p<0.05		p<0.05	p<0.05	
Up-regulated	**73**	56 (224)	53 (258)	44 (198)	49 (227)	**56**	45 (172)	53 (291)	**45**
Down-regulated	**64**	36 (119)	39 (154)	33 (98)	37 (150)	**39**	14 (48)	31 (164)	**13**

**Fig 1:**
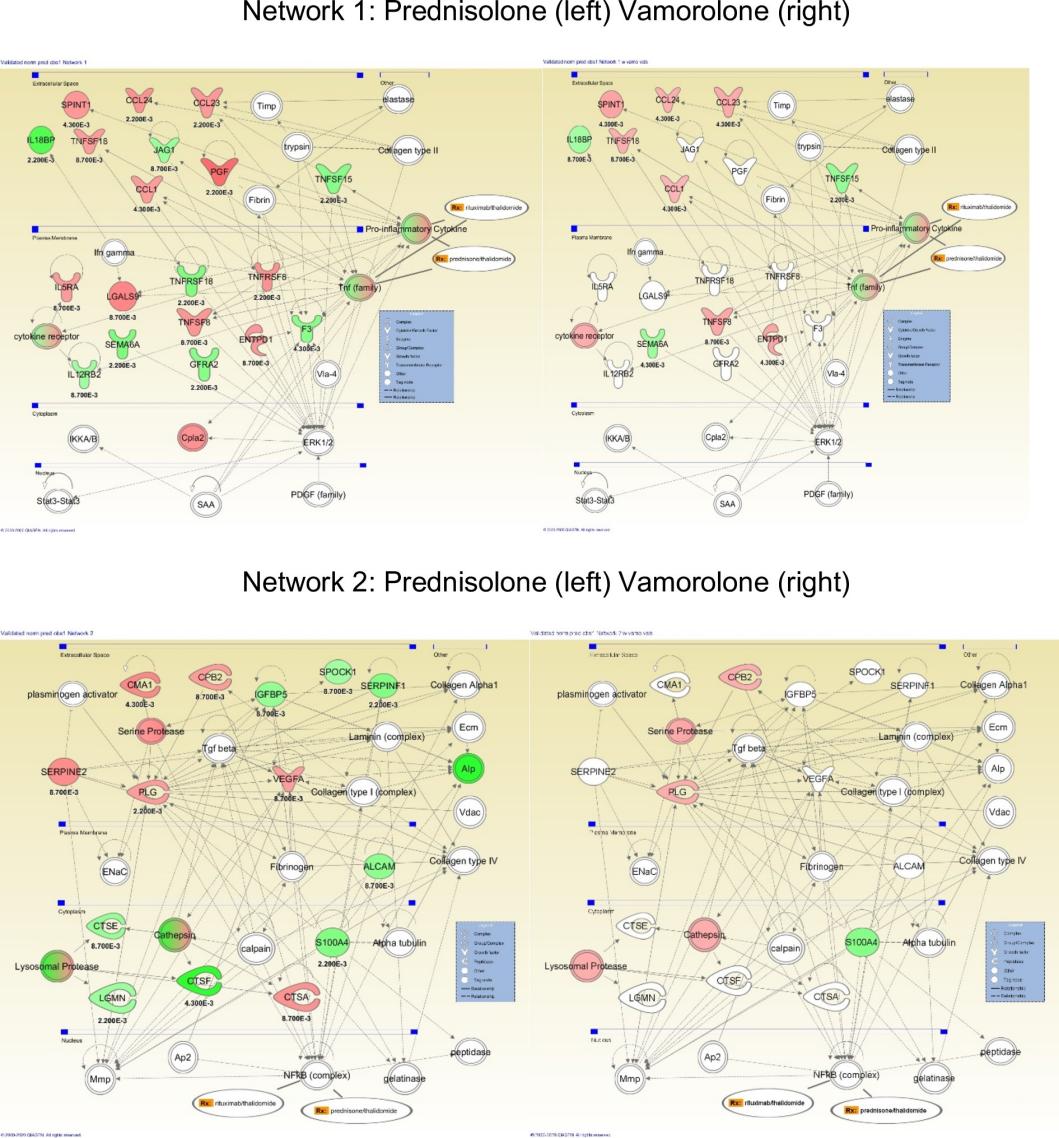
Ingenuity Pathway Analysis of prednisolone-responsive biomarkers identifies inflammatory cytokine and NFκB pathways shared with vamorolone. The two most significant enrichment of protein-protein interactions for prednisolone-responsive biomarkers are shown (left panels), with vamorolone-responsive proteins overlaid onto the prednisolone networks. Red indicates upregulation, and green indicates downregulation, and color intensity correlates with the size of expression change. The multicolored elements are protein complexes where the underlying data and documentation are inconsistent with IPAs search algorithms, and therefore, the details are not shown.

The 95 proteins concordant for 4 or more of the 5 prednisolone-treated groups tested were tested for biological connectivity using the Ingenuity Pathway Analysis (IPA) software. This software queries literature-supported functional associations between proteins and corresponding genes. The two top networks are shown using Entrez Gene Symbols corresponding to the SOMAscan protein IDs (**Fig 1**and **Table 3**). Network 1 was focused on TGFβ interacting proteins and genes, while Network 2 was focused on NFκB and tumor necrosis factor (TNF) interacting proteins and genes. For Network 1, there was upregulation of multiple C-C chemokine ligands (CCL1, CCL23, CCL24), lectin (LGALS9), and interleukin family member (IL5RA) and downregulation of interleukin family members (IL12RB2, IL18BP) and protein JAG-1. Network 2 showed upregulation of mast cell chymase (CMA1) and interleukin family members (IL5RA, IL12RB2, IL18RAp, IL18BRAP). TNF family members were variably upregulated (TNFSF8, TNFRSF8, TNFSF18) and downregulated (TNFSF15, TNFRSF18). Inducible T cell co-regulator (ICOSLG in network 3, not shown) was down-regulated. When no pharmacological agents were superimposed on the networks, IPA detected Network 2 as corticosteroid-responsive in the literature.

**S3 Fig in S1 File** shows IPA diagrams for all prednisolone groups (a) Prednisolone only, b) Prednisolone and Rituximab, c) Prednisolone plus BAIBA low dose, d) Prednisolone plus BAIBA high dose, e) Prednisolone plus Rituximab plus BAIBA low dose). There is very little change in these groups. Fold changes and p-values are listed for each protein in the diagram.

### Vamorolone-responsive serum biomarkers

Vamorolone is a first-in-class dissociative steroidal drug under development as a potential replacement for corticosteroids. Vamorolone is a partial agonist of the corticosteroid receptor and antagonist of the mineralocorticoid receptor (MR) (the latter in contrast to prednisolone, an MR agonist). As the mechanism of action of vamorolone is similar to that of prednisolone (binding to the corticosteroid receptor), we utilized the same prednisolone-responsive candidates. We tested them in the two vamorolone-treated groups (Groups 10, 11). This comparison showed that 13 down-regulated serum biomarkers were shared between prednisolone- and vamorolone-treated mice, and 45 up-regulated proteins (p<0.05 for both groups 10 and 11) and one protein (FSH) that showed opposite directionality between prednisolone and vamorolone. The serum proteins that were prednisolone-responsive and consistently responsive to vamorolone are shown individually with p-values in **S1a Table in S1 File**. The fold changes for these 59 proteins were plotted for the vamorolone validation group (G11). The plots are shown in **S1E Fig in S1 File**. Except for some potential outliers, these plots are unremarkable. A Venn diagram showing these results graphically is shown in **S2C Fig in S1 File**. All these proteins are listed in the S2 File.

Vamorolone protein data was overlaid with the prednisolone-responsive IPA Network 1 and Network 2 (**Fig 1**and **Table 4**). Of the 12 network members upregulated by prednisolone in Network 1, 8 were also upregulated by vamorolone but to a lesser extent. Similarly, of the 8 network members downregulated by prednisolone, 3 were downregulated by vamorolone. For Network 2, the proteins upregulated by prednisolone were similarly upregulated by vamorolone, whereas proteins downregulated by prednisolone were generally not shared by vamorolone treatment. This data suggests that the upregulated interleukin and TNF family members were most closely shared by vamorolone and prednisolone.

**Table 4:** Prednisolone-responsive serum proteins mapped to two IPA networks (Network 1 TGFβ; Network 2 NFκB and TNF), and correlation of vamorolone drug response.

Protein identifying information	Rituximab + prednisolone treatment group			BAIBA low dose + prednisolone treatment group			BAIBA high dose + prednisolone treatment group			BAIBA low dose + Rituximab + prednisolone			Vamorolone			Vamorolone + BAIBA low dose + Rituximab treatment group		
	N	P-value (comparison to vehicle)	Fold Change (comparison to vehicle)	N	P-value (comparison to vehicle)	Fold Change(comparison to vehicle)	N	P-value (comparison to vehicle)	Fold Change(comparison to vehicle)	N	P-value (comparison to vehicle)	Fold Change(comparison to vehicle	N	P-value (comparison to vehicle)	Fold Change(comparison to vehicle	N	P-value (comparison to vehicle)	Fold Change(comparison to vehicle)
**UP-REGULATED**																		
Cathepsin A (CTSA; P10619)	6	0.0081	1.191	6	0.0043	1.355	6	ns	ns	6	0.0022	1.337	6	0.026	1.152	6	0.026	1.798
CD30 (TNFRSF8; P28908)	6	0.0043	1.184	6	0.0087	1.57	6	0.026	1.133	6	0.0043	1.266	6	ns	ns	6	0.0087	1.123
CD30 Ligand (TNFSF8; P32971)	6	0.0087	1.279	6	0.0411	1.62	6	0.0087	1.327	6	0.026	1.467	6	0.0087	1.25	6	0.0022	1.452
CD39 (ENTPD1; P49961)	6	0.0043	1.288	6	0.0152	1.559	6	0.0022	1.372	6	0.0043	1.619	6	0.0043	1.298	6	0.0022	1.376
Chymase (CMA1; P23946)	6	0.026	1.152	6	0.0152	1.709	6	0.026	1.195	6	ns	ns	6	ns	ns	6	0.0087	1.271
Ck-b-8-1 (CCL23; P55773)	6	0.0022	1.217	6	0.0087	1.246	6	0.0087	2.639	6	0.0087	2.128	6	0.0043	1.225	6	0.0022	1.186
Eotaxin-2 (CCL24; O00175)	6	0.0022	1.208	6	0.0152	1.304	6	0.0022	1.163	6	0.0022	1.264	6	0.0043	1.136	6	0.0022	1.219
HAI-1 (SPINT1; O43278)	6	0.0043	1.321	6	0.0087	1.381	6	0.0022	1.344	6	0.0087	1.46	6	0.0043	1.317	6	0.0022	1.391
**Protein**	**N**	**G6 p-val**	**G6 FC**	**N**	**G7 p-val**	**G7 FC**	**N**	**G8 p-val**	**G8 FC**	**N**	**G9 p-val**	**G9 FC**	**N**	**G10 p-val**	**G10 FC**	**N**	**G11 p-val**	**G11 FC**
I-309 (CCL1; P22362)	6	0.0022	1.176	6	0.0043	1.536	6	ns	ns	6	0.0022	1.343	6	0.0043	1.082	6	0.0152	1.13
IL-5 Ra (IL5RA; Q01344)	6	0.0087	1.2	6	0.0087	1.408	6	0.0152	1.168	6	0.0152	1.295	6	0.026	1.161	6	0.0081	1.215
LEG9 (LGALS9; O00182)	6	0.0087	1.248	6	0.026	1.646	6	0.026	1.211	6	ns	ns	6	ns	ns	6	0.0087	1.258
PLGF (PGF; P49763)	6	0.0022	1.236	6	0.0303	1.258	6	ns	ns	6	0.0022	1.318	6	0.0411	1.073	6	0.0411	1.14
Plasminogen (PLG; P00747)	6	0.0022	1.247	6	0.0152	1.382	6	0.0022	1.242	6	0.026	1.299	6	0.0087	1.195	6	0.0081	1.228
Protease nexin I (SERPINE2; P07093)	6	0.0411	1.238	6	0.0411	2.367	6	0.026	1.317	6	ns	ns	6	ns	ns	6	0.0022	1.478
TAFI (CPB2; Q96IY4)	6	0.0087	1.193	6	0.0161	1.484	6	ns	ns	6	0.045	1.26	6	0.0043	1.163	6	0.0022	1.24
TNFSF18 (TNFSF18; Q9UNG2)	6	0.0022	1.254	6	0.0087	1.429	6	0.0087	1.198	6	0.0152	1.272	6	0.0087	1.141	6	0.0022	1.228
VEGF (VEGFA; P15692)	6	0.0043	1.2	6	ns	ns	6	0.0022	1.306	6	0.0087	1.207	6	0.0152	1.16	6	0.0152	1.142
DOWN-REGULATED																		
ALCAM (ALCAM; Q13740)	6	0.0087	-1.275	6	0.0087	-1.87	6	ns	ns	6	0.0411	-1.336	6	ns	ns	6	0.0087	-1.291
CATE (CTSE; P14091)	6	0.0152	-1.225	6	0.0087	-1.428	6	ns	ns	6	0.0411	-1.346	6	ns	ns	6	0.0152	-1.272
**Protein**	**N**	**G6 p-val**	**G6 FC**	**N**	**G7 p-val**	**G7 FC**	**N**	**G8 p-val**	**G8 FC**	**N**	**G9 p-val**	**G9 FC**	**N**	**G10 p-val**	**G10 FC**	**N**	**G11 p-val**	**G11 FC**
CATF (CTSF; Q9UBX1)	6	0.0022	-2.653	6	0.0022	-3.424	6	0.0087	-2.256	6	ns	ns	6	0.0022	-2.682	6	ns	ns
GFRa-2 (GFRA2; O00451)	6	0.0022	-1.507	6	0.0087	-1.322	6	0.0022	-1.421	6	0.0022	-1.518	6	0.0411	-1.163	6	0.0022	-1.395
GITR (TNFRSF18; Q9Y5U5)	6	0.0043	-1.427	6	0.026	-1.98	6	0.0043	-1.521	6	0.0022	-1.653	6	ns	ns	6	0.0087	-1.323
IGFBP-5 (IGFBP5; P24593)	6	0.0022	-1.356	6	0.0087	-1.873	6	0.0022	-1.277	6	0.0022	-1.57	6	0.0152	-1.273	6	0.0022	-1.434
IL-12 RB2 (IL12RB2; Q99665)	6	0.0411	-1.184	6	0.0087	-1.447	6	0.0411	-1.213	6	0.026	-1.308	6	ns	ns	6	ns	ns
IL-18 BPa (IL18BP; O95998)	6	0.0022	-1.813	6	0.0022	-2.585	6	0.0022	-1.736	6	0.0022	-1.935	6	0.0087	-1.215	6	0.0022	-1.527
JAG1 (JAG1; P78504)	6	0.0087	-1.293	6	0.0022	-1.75	6	ns	ns	6	0.0152	-1.386	6	ns	ns	6	0.0087	-1.303
LGMN (LGMN; Q99538)	6	0.0022	-1.316	6	0.0022	-1.472	6	0.0022	-1.311	6	0.0022	-1.448	6	0.026	-1.228	6	0.0087	-1.386
PEDF (SERPINF1; P36955)	6	0.0022	-1.425	6	0.0022	-2.025	6	0.0022	-1.395	6	0.0022	-1.61	6	ns	ns	6	0.0152	-1.235
Semaphorin-6a (SEMA6A; Q9H2E6)	6	0.0043	-1.472	6	0.0022	-2.212	6	0.0043	-1.473	6	0.0022	-1.829	6	0.0043	-1.321	6	0.0022	-1.49
S100A4 (S100A4; P26447)	6	0.0022	-1.701	6	0.0152	-1.942	6	0.0022	-1.613	6	0.0411	-1.741	6	0.0022	-1.588	6	0.026	-1.647
Testican-1 (SPOCK1; Q08629)	6	ns	ns	6	0.0152	-1.385	6	0.026	-1.19	6	0.026	-1.299	6	0.026	-1.15	6	0.0022	-1.211
TF (F3; P13726)	6	0.0152	-1.317	6	0.0043	-1.925	6	0.0087	-1.3	6	0.0411	-1.428	6	ns	ns	6	0.0411	-1.253
**Protein**	**N**	**G6 p-val**	**G6 FC**	**N**	**G7 p-val**	**G7 FC**	**N**	**G8 p-val**	**G8 FC**	**N**	**G9 p-val**	**G9 FC**	**N**	**G10 p-val**	**G10 FC**	**N**	**G11 p-val**	**G11 FC**
TNFSF15 (TNFSF15; O95150)	6	0.0022	-1.572	6	0.0022	-1.528	6	0.0022	-1.534	6	0.0022	-1.576	6	0.0022	-1.383	6	0.0022	-1.371

Yellow highlights are Network 1 members, and blue highlights Network, 2 members. ns indicates “not significant.

**S6C and S6D Fig in S1 File** show IPA diagrams for the two vamorolone groups (c) Vamorolone only, d) Vamorolone plus rituximab plus BAIBA low dose.) As is explained above, the vamorolone and prednisolone IPA network 1 diagram is very similar. This is to be expected based on prednisolone and vamorolone work in very similar ways with respect to anti-inflammatory properties. Fold changes and p-values are listed for each.

### Comparison to human corticosteroid-responsive serum biomarkers

Thirty-seven (37) serum proteins have been reported to be responsive to corticosteroids in human studies of DMD and pediatric inflammatory bowel disease. These were aligned with the *mdx* mouse data (**S4 Table in S1 File**) [15, 16, 27] Fourteen of 37 (38%) showed signals in the *mdx* mouse studies that were <110 relative fluorescence units on the SOMAscan assay, suggesting poor cross-reactivity. One of these 14 (7%) (TECK) showed statistical significance in 3 or more *mdx* treatment groups. Of the remaining 23 human steroid-responsive serum proteins, eight showed significance in 3 or more *mdx* comparison groups (34.8%) (Afamin, CD23, CD36 Antigen, MPIF-1, Resistin, SLIK5, TECK, and, Testican-2). One of these proteins (TECK) had an RFU value (85.2) well below 110 leaving seven candidates. Four of the seven (Afamin, CD36 Antigen, SLIK5, and Testican-2) however, showed the same directionality between human and mouse, and three (CD23, MPIF-1, and Resistin) showed opposite directionality (e.g., decreased in human, and increased in *mdx* mouse; e.g., CCL23 was down-regulated by corticosteroid treatment in IBD but upregulated in *mdx*). Of the four discordant for directionality, two were from human DMD data (FCER-2, SLIK-5), and two from human IBD data (MPIF-1, Resistin).

### Rituximab-responsive serum biomarkers

Rituximab is an anti-CD20 antibody that depletes B cells. A similar “discovery” and “validation” approach was used to identify Rituximab-responsive serum biomarkers, with the “discovery” comparison being Group 1 (vehicle) vs. Group 3 (rituximab alone), and three validation data sets (Group 1 vs. Groups 6, 9, 11) (**Table 5**). These were not independent validations, as the comparisons to the later groups were against the same vehicle control (Group 1).

**Table 5:** Serum proteomics comparisons of drug-treated vs. non-treated controls (Group 1) for rituximab-treated *mdx* mice.

	Group 3 rituximab	Group 6 Prednisolone + Rituximab	Group 9 Prednisolone + βAIBA (low dose) + Rituximab	Group 11 Vamorolone + βAIBA (low dose) + Rituximab	Rituximab concordant (3 of 3 groups)
	P<0.01	P<0.05	p<0.05	p<0.05	
Group 1 vs Upregulated	**13**	5 (224)	6 (227)	6 (291)	**4**
Group 1 vs. down-regulated	**12**	1 (119)	0 (150)	2 (164)	**0**

Comparisons of the vehicle (Group 1) vs. rituximab alone (Group 3) showed 25 proteins with p<0.01. Our statistical analysis plan then tested each of these candidates as pairwise comparisons in Groups 6, 9, and 11, where each biomarker needed to show p<0.05 (**Table 5**). The analysis showed 13 proteins consistently up-regulated by rituximab alone, and 12 proteins consistently down-regulated by rituximab alone. After the pairwise comparisons to groups 6, 9, and 11, only 4 proteins were consistently upregulated, and 0 proteins consistently down-regulated. Serum proteins that were consistently rituximab–responsive are shown individually with p values (**Table 6**). The fold changes for the 25 proteins were plotted for the rituximab validation groups (G6, G9, and G11). The plots are shown in **S1B Fig in S1 File**. Rituximab plots seem compressed to the lower-left due to large outliers. Rituximab is a chimeric anti-CD20 monoclonal antibody with mouse variable and human constant regions. IgG (human) was detecting the humanized antibody used as the treatment drug (rituximab) and accounts for one of the major outliers. A Venn diagram showing these results graphically is shown in **S2B Fig in S1 File**. All these proteins are listed in the S2 File.

**Table 6:** Summary of drug-responsive biomarkers for rituximab.

Protein Identifying Information	Uniprot ID	RituximabTtreatment Group (Group 3)			Rituximab + Prednisolone Treatment Group (Group 6)			BAIBA low dose + Rituximab + Prednisolone Treatment Group (Group 9)			Vamorolone + BAIBA low dose + Rituximab Treatment Group (Group 11)		
		n	P-value (comparison to vehicle)	Fold Change(comparison to vehicle)	n	P-value (comparison to vehicle)	Fold Change(comparison to vehicle)	n	P-value (comparison to vehicle)	Fold Change(comparison to vehicle)	n	P-value (comparison to vehicle)	Fold Change(comparison to vehicle)
IgG	P01857	6	0.0022	25.018	6	0.0152	29.84	6	0.0152	37.654	6	0.0022	37.316
NADPH-P450 Oxidoreductase	P16435	6	0.0087	1.334	6	0.0043	1.448	6	0.0043	1.56	6	0.0043	1.496
ADAM 9	Q13443	6	0.0087	1.602	6	0.0043	1.273	6	0.0087	1.496	6	0.0043	1.271
Plasmin	P00747	6	0.0087	1.205	6	0.0022	1.247	6	0.026	1.299	6	0.0081	1.228

Shown are proteins that were significant by Group 1 (control) vs. Group 3 (rituximab) and then also tested as significant in the three other group comparisons (p<0.05; Group 1 vs. Groups 6, 9, or 11).

**S4 Fig in S1 File** shows IPA diagrams for all rituximab groups (a) Rituximab b) Rituximab plus prednisolone, c) Rituximab plus prednisolone plus BAIBA low dose, d) Rituximab plus vamorolone plus BAIBA low dose. There is very little change throughout the groups. Since rituximab is a monoclonal antibody for B-lymphocyte, CD20 receptors which mediate B cell depletion and death, the other three molecules (Adam 9, PLG, and POR) are two peptidases and an oxidoreductase are used in breaking down cellular components and would be consistent with cellular death. None of the other drugs would cause CD20 induced apoptosis. Fold changes and p-values are listed for each protein in the diagram.

### BAIBA-responsive serum biomarkers

BAIBA low dose (100 mg/kg/day) was administered to 4 different groups of mice, either alone (Group 4) or in combination with other drugs (Groups 7, 9, 11) (**Table 7**). As noted in the other treatment groups, the number of mice in each group (n = 6) was insufficient to adjust for multiple testing of 1,310 protein tests statistically; therefore, a ‘discovery’ data set (vehicle [Group 1] vs. BAIBA low dose [Group 4]), with an unadjusted p-value threshold p<0.01. Serum proteins that were significantly changed by BAIBA low dose alone were tested as ‘candidates’ in the other groups that included BAIBA treatment (Groups 7, 9, 11). Validations were not independent, as the later groups’ comparisons were against the same vehicle control (Group 1).

**Table 7:** Serum proteomics comparisons of drug-treated vs. non-treated controls (Group 1) for BAIBA-treated *mdx* mice.

	Group 4 βAIBA (low dose)	Group 7 Prednisolone + βAIBA (low dose)	Group 9 Prednisolone + βAIBA (low dose) + Rituximab	Group 11 Vamorolone + βAIBA (low dose) + Rituximab	BAIBA low dose Concordance	Group 5 βAIBA (high dose)	Group 8 Prednisolone + βAIBA (high dose)	BAIBA High dose Concordance
	P<0.01	P<0.05	p<0.05	p<0.05		P<0.01	P<0.05	
Group 1 vs Upregulated	**11**	6 (258)	6 (227)	6 (291)	**6**	44	34 (198)	**34**
Group 1 vs. down-regulated	**32**	4 (154)	4 (150)	4 (164)	**4**	7	1 (98)	**1**

BAIBA high dose (500 mg/kg/day) was administered to 2 different groups of mice, either alone (Group 5) or in combination with other drugs (Group 8) (**Table 1**). A ‘discovery’ data set (vehicle [Group 1] vs. BAIBA high dose [Group 4]), with an unadjusted p-value threshold p<0.01 was used. Serum proteins that were significantly changed by BAIBA high dose alone were then tested as ‘candidates’ in the other group that included BAIBA treatment (Group 8). These were not independent validations, as the comparisons to the later groups were against the same vehicle control (Group 1).

The results showed 11 proteins up-regulated and 32 proteins down-regulated by BAIBA low dose alone. After the pairwise comparisons to groups 7, 9, and 11, 6 proteins were consistently upregulated, and 4 proteins were consistently down-regulated. Serum proteins consistently BAIBA low dose-responsive are shown individually with p values (**S2 Table in S1 File**).

The results also showed 44 proteins up-regulated and 7 proteins down-regulated by BAIBA high dose alone. After the pairwise comparisons to group 8, 34 proteins were consistently upregulated, and 1 protein was consistently down-regulated. Serum proteins consistently BAIBA high dose-responsive are shown individually with p values in (**S3 Table in S1 File**). The fold changes for the 43 proteins were plotted for the BAIBA low dose validation groups (G7, G9, and G11). The plots are shown in **S1C Fig in S1 File**. Except for some potential outliers, these plots show nothing remarkable. A Venn diagram showing these results graphically is shown in **S2D Fig in S1 File**. Based on the numbers of proteins involved, the proteins are detailed in the S2 File.

**S5 Fig in S1 File** shows IPA diagrams for all BAIBA low dose groups (a) BAIBA low dose, b) BAIBA low dose plus prednisolone, c) BAIBA low dose plus rituximab plus prednisolone, d) BAIBA low dose plus rituximab plus vamorolone). There is very little change throughout the groups. Fold changes and p-values are listed for each protein in the diagram.

**Fig 2:**
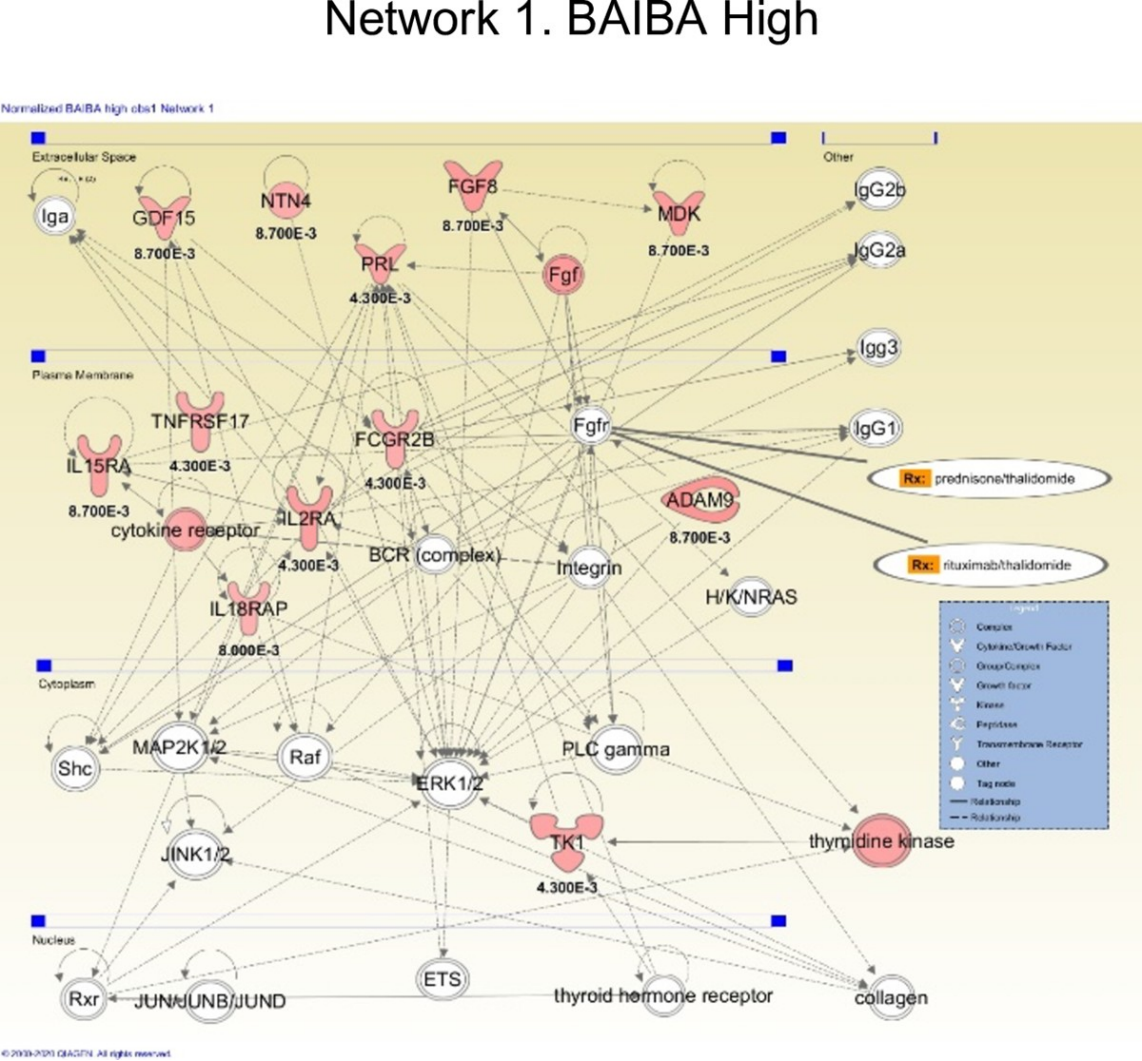
Ingenuity Pathway Analysis of BAIBA-responsive biomarkers identifies modulation of inflammatory pathways. The top IPA network identified with high dose BAIBA treatment data is shown.

The serum proteins responsive to high dose BAIBA were tested with IPA software for networks, and the most likely top network was developed (**Fig 2**). High dose BAIBA responsive proteins were centered on an inflammatory pathway involving upregulation of two anti-inflammatory proteins (TYRO3, a potent negative regulator of inflammation; and IL2RA, loss of function leads to autoimmunity in humans), upregulation of a receptor involved in inducing apoptosis (TNFRSF10B), and upregulation of cell adherence proteins (CADM1, CADM2). **S6A and S6B Fig in S1 File** show IPA diagrams for the two BAIBA high dose groups (a) BAIBA high dose, b) BAIBA high dose plus prednisolone). There is very little change throughout the groups. Fold changes and p-values are listed for each protein in the diagram.

For further exploration in another study, we also identified that BAIBA affected proteins such as NADPH-P450 Oxidoreductase, GDF15, FGF-8B, and PRL that are known to play a role in the growth and metabolism of several cell types (Please see **S3 Table in S1 File** for a complete list and **Fig 2**).

## Discussion

DMD has four cellular and tissue pathology hallmarks; muscle cell membrane damage [9, 21], loss of metabolic patency [11–13], inflammatory response [21, 23], and fibrosis [2]. With multiple pathology hallmarks, any successful treatment will likely consist of a multi-drug approach, with each cellular hallmark targeted by specific drugs. It is also likely true that dystrophin replacement therapies (exon skipping, gene therapy) will require a combination therapy approach. All four cellular pathologies persist to some degree even after semi-functional dystrophin is introduced.

Multi-drug approaches are difficult to develop pre-clinically and clinically, as it can be challenging to parse the effects of each drug on histological or clinical outcomes. We hypothesized that a short-term pre-clinical study of serum biomarkers might provide acute and objective outcome measures for testing and optimizing multi-drug approaches to DMD, and tested this hypothesis using the *mdx* mouse model, with four drugs singly and in combination (prednisolone, vamorolone, rituximab, BAIBA). Prednisolone, vamorolone, and BAIBA all target inflammation via suppression of NFκB-related pro-inflammatory pathways. Vamorolone also targets membrane instability [9], and BAIBA also targets cell metabolism. Rituximab targets B cells and inflammatory cascades. Rituximab is known to bind to alternative targets such as sphingomyelinase-like phosphodiesterase 3b (SMPDL3B) expressed in many cell types and is a modulator of insulin signaling [28].

We hypothesized that beneficial effects of these drugs in the *mdx* mouse model of DMD might be complementary, and methods to monitor complementary effects might be developed using serum biomarker studies.

The tool used to evaluate the serum biomarkers was SOMAscan (SOMAlogic, Inc.). This assay returns values in relative fluorescent units that correspond to the relative levels of 1,310 serum proteins detected using specific aptamers (SOMAmers) [29]. The SOMAmers used in this assay were optimized for the detection of human serum proteins. This clearly led to limitations of our approach, as the cross-reactivity of the human aptamers to murine serum proteins is not well-defined. While there is a previous report of utilizing the SOMAscan assay with mouse serum samples, [30] the sensitivity and specificity of the SOMAscan assay on the mouse serum proteins is questioned.

There were 11 groups of mice in this study, with each group having mean and standard deviation values for all 1,310 proteins. All groups were compared (by the Mann-Whitney rank-sum test) to at least one (vehicle) group. Based on many comparisons being performed, the effect of multiple testing needed to be addressed. The analysis compared 1,310 values at a time. Since the group sizes were quite small (n = 6), a large-scale correction for multiple testing such as the Bonferroni method would be too stringent and would have likely led to none of the proteins being flagged as significant. To account for the expected large number of false positives that would result from not accounting for multiple testing, a “discovery” and “validation” approach was used. In the prednisolone “discovery” phase, comparisons between prednisolone and vehicle were considered significant if they had a p-value less than 0.01. This reduced the number of positive results that may have happened by chance to approximately 13. The “validation” phase consisted of comparing other groups of mice that had been treated with prednisolone in addition to other drugs to the vehicle group using only the significant findings from the discovery phase. The intent was although the additional treatments may affect the levels measured in these mice compared to the prednisolone only treated mice, prednisolone should still dominate the overall results because of its strength as an anti-inflammatory. Four other groups were analyzed, with significance determined at a value of p<0.05. If three (or four) of these validation groups also showed significance, they were used in the next analysis. This group of results was considered our "data mask" used for further analysis. Using this approach, any false positives that survived the discovery phase should have been found and removed by the validation stage. It needs to be noted that these were not independent validations, as the comparisons to the later groups were against the same vehicle control (Group 1), which was another limitation of this study. This process yielded 95 significant proteins (56 up-regulated, 39 down-regulated) in the prednisolone vs. vehicle test. The pharmacodynamic biomarkers detected were focused on NFκB and TFGβ pathways, as expected (**Fig 1**).

Corticosteroid-responsive serum biomarkers in human DMD and pediatric inflammatory bowel disease patients have been previously reported using the same SOMAscan platform [15, 27, 31]. We aligned the 37 previously reported human pharmacodynamic biomarkers with the *mdx* mouse data (**S4 Table in S1 File**). This showed that 14 of the 37 had very low signals in mouse sera, suggestive of poor cross-reactivity of human aptamer probes in the mouse sera. Of the 23 with stronger signals, 13% (3/23) showed concordance between mouse and human (SLITRK5, AFM, and FCER2). These three biomarkers hold promise as corticosteroid-responsive biomarkers that cross-species boundaries.

Vamorolone and prednisolone share a similar structure and both bind with high affinity to the corticosteroid receptor. However, they show differential downstream activities. To evaluate the effects of vamorolone compared to prednisolone on serum biomarkers in the *mdx* mouse, the two vamorolone groups were compared to the vehicle group at p<0.05, and the results were compared to the prednisolone data mask. This resulted in 45 common up-regulated proteins and 13 common down-regulated proteins. This data is consistent with some shared downstream activities of vamorolone vs. prednisolone. This also suggests that prednisolone has a more pervasive effect on the serum proteins; this might also be expected since vamorolone shows less genomic (gene transcriptional) effects than corticosteroids [7, 21, 32].

To evaluate the effects of rituximab and BAIBA, the same “discovery” and “validation” approach was used. Rituximab was compared to a vehicle with a significance of p<0.01. The proteins that survived this level were compared to the three other treatment groups using rituximab. In this case, all groups needed to have p<0.05 to pass this validation. Only four up-regulated proteins survived this validation (**Table 5**). An interesting observation is that the protein IgG showed huge up-regulation values. The SOMAmers are designed to respond to human targets, and rituximab is a human monoclonal antibody. Since a mouse would not naturally have human IgG, the initial or vehicle number would be very low (ideally zero). When rituximab was administered to the mice, SOMAscan detected this as IgG. This detection would register as a relatively large value compared to the vehicle number leading to the high fold change. The high fold change was observed and could be considered a positive experimental control in this study.

The same approach was taken for BAIBA high dose and low dose. BAIBA low dose resulted in 43 significant proteins (11 up-regulated and 32 down-regulated), and BAIBA high dose resulted in 51 significant proteins (44 up-regulated and 7 down-regulated) (**Table 7**; **S3 Table in S1 File**). Of the 10 validated BAIBA low dose proteins, and the 35 validated BAIBA high dose proteins, 1 protein (SLAF6) was validated in both sets. Most serum biomarkers for BAIBA appeared dose-responsive, and IPA networks centered on tyrosine kinases and anti-inflammatory proteins’ upregulation (TYRO3; IL2RA) (**Fig 2**).

In summary, our data indicate that prednisolone effects either alone or in combination with other drugs have profound effects on body weight gain and serum biomarkers. As expected, prednisolone shared NFkB-anti-inflammatory (e.g., CCL23, CCL-1, TNFSF18) but not GRE mediated gene products (e.g., PGF, CPLA2) with vamorolone. BAIBA affected both inflammatory and metabolic proteins. Rituximab did not significantly alter serum makers, probably due to lower cross-reactivity to human CD20. Our data suggests that a serum biomarker approach has promise in pre-clinical and clinical testing in combination therapies. We found concordance of data between different treatment groups where a single drug was held in common, and some overlap with human pharmacodynamic biomarkers for the same drug. This study’s limitations included concerns regarding cross-reactivity of human aptamer probes to mouse serum proteins, the use of a single “combined placebo” control group, and potential challenges in translating the murine findings to human clinical trials.

## Supporting information

S1 File (PDF)

S2 File (XLSX)
