# Supplementary material for: Biomarker-focused multi-drug combination therapy and repurposing trial in mdx mice
Source: PLoS One. 2021 Feb 22;16(2):e0246507. doi: 10.1371/journal.pone.0246507 (PMC7899329; doi:10.1371/journal.pone.0246507)
Supplement: S1 File — (PDF) [file pone.0246507.s001.pdf]

Supplementary Information for Multidrug AGADA Trial

S1 File

Supplementary Table 1. Summary of candidate efficacy and safety biomarkers for vamorolone and prednisolone. Shown are proteins that were significant by Group 1 (vehicle) vs. Group 2 (prednisolone), and then also tested as significant in three of the four validation group comparisons (p<0.05; Group 1 vs. Groups 6, 7, 8, or 9). ns indicates "not significant"

Protein Identifying Information	Uniprot ID	Prednisolone (Group 2)			Rituximab + Prednisolone Treatment Group (Group 6)			BAIBA low dose + Prednisolone Treatment Group (Group 7)			BAIBA high dose + Prednisolone Treatment Group (Group 8)			BAIBA low dose + Rituximab + Prednisolone Treatment Group (Group 9)		
		n	P-value (comparison to vehicle)	Fold Change(comparison to vehicle)	n	P-value (comparison to vehicle)	Fold Change(comparison to vehicle)	n	P-value (comparison to vehicle)	Fold Change(comparison to vehicle)	n	P-value (comparison to vehicle)	Fold Change(comparison to vehicle)	n	P-value (comparison to vehicle)	Fold Change(comparison to vehicle)
14-3-3 protein zeta/delta	P63104	6	0.0087	-1.392	6	0.026	-1.312	6	0.0152	-1.667	6	0.0411	-1.246	6	0.0087	-1.463
AGR2	O95994	6	0.0081	1.206	6	0.005	1.272	6	0.0081	1.392	6	0.02	1.239	6	0.0303	1.329
ALCAM	Q13740	6	0.0087	-1.504	6	0.0087	-1.275	6	0.0087	-1.87	6	ns	ns	6	0.0411	-1.336
Alkaline phosphatase, bone	P05186	6	0.0022	-2.811	6	0.0022	-2.654	6	0.0022	-3.713	6	0.026	-2.262	6	0.0411	-2.438
Apo A-I	P02647	6	0.0087	1.44	6	0.0152	1.315	6	0.0152	1.93	6	ns	ns	6	0.026	1.537
ARSB	P15848	6	0.0087	1.191	6	0.0043	1.221	6	0.0152	1.354	6	0.0087	1.179	6	0.026	1.402
AURKB	Q96GD4	6	0.0087	1.221	6	0.0411	1.143	6	0.0411	1.417	6	ns	ns	6	0.0411	1.206
Aurora kinase A	O14965	6	0.0087	1.592	6	0.0152	1.321	6	0.0152	1.644	6	0.026	1.432	6	ns	ns
B7-H2	O75144	6	0.0022	-1.173	6	0.0022	-1.185	6	0.0043	-1.436	6	0.026	-1.163	6	ns	ns
BCL2-like 1 protein	Q07817	6	0.0043	1.288	6	0.0043	1.291	6	0.0043	1.665	6	0.026	1.185	6	0.0087	1.506
bFGF-R	P11362	6	0.0022	-1.568	6	0.0043	-1.364	6	0.0043	-1.947	6	0.026	-1.274	6	0.0022	-1.526
BGH3	Q15582	6	0.0087	-1.564	6	0.026	-1.397	6	0.026	-2.064	6	0.0087	-1.369	6	0.0411	-1.454
C34 gp41 HIV Fragment	Q70626	6	0.0022	1.671	6	0.0022	1.676	6	ns	ns	6	0.0022	1.586	6	0.0152	1.464
Cadherin-5	P33151	6	0.0022	-2.122	6	0.0022	-1.847	6	0.0022	-2.647	6	0.0022	-1.712	6	0.0022	-1.907

Supplementary Table 1 - continued

**Protein**	**Uniprot**	**n**	**G2 p-val**	**G2 FC**	**n**	**G6 p-val**	**G6 FC**	**n**	**G7 p-val**	**G7 FC**	**n**	**G8 p-val**	**G8 FC**	**n**	**G9 p-val**	**G9 FC**
CATE	P14091	6	0.0087	-1.408	6	0.0152	-1.225	6	0.0087	-1.428	6	ns	ns	6	0.0411	-1.346
CATF	Q9UBX1	6	0.0043	-2.782	6	0.0022	-2.653	6	0.0022	-3.424	6	0.0087	-2.256	6	ns	ns
Cathepsin A	P10619	6	0.0087	1.305	6	0.0081	1.191	6	0.0043	1.355	6	ns	ns	6	0.0022	1.337
CD23	P06734	6	0.0022	1.377	6	0.0022	1.351	6	0.026	1.736	6	0.0043	1.351	6	0.026	1.641
CD30	P28908	6	0.0022	1.335	6	0.0043	1.184	6	0.0087	1.57	6	0.026	1.133	6	0.0043	1.266
CD30 Ligand	P32971	6	0.0087	1.272	6	0.0087	1.279	6	0.0411	1.62	6	0.0087	1.327	6	0.026	1.467
CD39	P49961	6	0.0087	1.24	6	0.0043	1.288	6	0.0152	1.559	6	0.0022	1.372	6	0.0043	1.619
CD83	Q01151	6	0.0022	-1.274	6	ns	ns	6	0.0152	-1.285	6	0.0247	-1.203	6	0.0411	-1.135
CDK2/cyclin A	P24941<br>P20248	6	0.0022	-1.677	6	0.0022	-1.669	6	0.0087	-1.633	6	0.0022	-1.867	6	0.0022	-1.79
Chitotriosidase-1	Q13231	6	0.0087	1.377	6	0.0087	1.295	6	0.026	1.588	6	0.0022	1.356	6	0.026	1.496
Chymase	P23946	6	0.0043	1.447	6	0.026	1.152	6	0.0152	1.709	6	0.026	1.195	6	ns	ns
Ck-b-8-1	P55773	6	0.0022	1.227	6	0.0022	1.217	6	0.0087	1.246	6	0.0087	2.639	6	0.0087	2.128
CK-MB	P12277<br>P06732	6	0.0043	-2.402	6	0.0022	-2.604	6	0.0087	-2.476	6	0.0043	-2.128	6	0.0152	-2.361
c-Myc	P01106	6	0.0043	1.498	6	0.0043	1.127	6	ns	ns	6	0.0411	1.11	6	0.0087	1.322
DLL1	O00548	6	0.0022	-1.789	6	0.0022	-1.638	6	0.0043	-2.107	6	0.0022	-1.66	6	0.0022	-1.66
DRAK2	O94768	6	0.0087	1.43	6	0.0152	1.362	6	0.026	1.764	6	0.0087	1.42	6	0.0411	1.555
Dtk	Q06418	6	0.0043	1.204	6	0.0043	1.322	6	0.0087	1.334	6	0.0043	1.349	6	0.0043	1.481
EGF	P01133	6	0.0087	1.656	6	0.0022	1.277	6	0.026	1.721	6	0.0087	1.307	6	ns	ns
Eotaxin-2	O00175	6	0.0022	1.329	6	0.0022	1.208	6	0.0152	1.304	6	0.0022	1.163	6	0.0022	1.264

Supplementary Table 1 - continued

Protein	Uniprot	n	G2 p-val	G2 FC	n	G6 p-val	G6 FC	n	G7 p-val	G7 FC	n	G8 p-val	G8 FC	n	G9 p-val	G9 FC
EPHB2	P29323	6	0.0022	-1.387	6	0.0087	-1.282	6	0.0022	-1.554	6	0.0022	-1.236	6	0.0087	-1.311
EphB6	O15197	6	0.0087	-1.46	6	0.0152	-1.34	6	0.0087	-1.649	6	ns	ns	6	0.0087	-1.406
FGF-17	O60258	6	0.0049	1.111	6	0.005	1.169	6	0.0303	1.164	6	ns	ns	6	0.0303	1.269
FLRT3	Q9NZU0	6	0.0087	-1.374	6	0.0087	-1.299	6	0.026	-1.566	6	0.0087	-1.367	6	0.026	-1.403
FSTL1	Q12841	6	0.0087	-1.556	6	ns	ns	6	0.0152	-2.032	6	0.0152	-1.486	6	0.0087	-1.776
GFRa-2	O00451	6	0.0022	-1.578	6	0.0022	-1.507	6	0.0087	-1.322	6	0.0022	-1.421	6	0.0022	-1.518
GITR	Q9Y5U5	6	0.0022	-1.691	6	0.0043	-1.427	6	0.026	-1.98	6	0.0043	-1.521	6	0.0022	-1.653
Glypican 3	P51654	6	0.0087	1.882	6	0.026	1.523	6	0.0152	2.479	6	0.0152	1.54	6	ns	ns
HAI-1	O43278	6	0.0043	1.258	6	0.0043	1.321	6	0.0087	1.381	6	0.0022	1.344	6	0.0087	1.46
HSP 90b	P08238	6	0.0087	-1.53	6	0.0087	-1.469	6	0.026	-1.767	6	0.026	-1.419	6	0.0043	-1.659
HXK1	P19367	6	0.0087	1.706	6	0.0152	1.227	6	0.0152	2.254	6	ns	ns	6	0.0411	2.095
I-309	P22362	6	0.0043	1.21	6	0.0022	1.176	6	0.0043	1.536	6	ns	ns	6	0.0022	1.343
IGFBP-5	P24593	6	0.0087	-1.424	6	0.0022	-1.356	6	0.0087	-1.873	6	0.0022	-1.277	6	0.0022	-1.57
IL-12 RB2	Q99665	6	0.0087	-1.275	6	0.0411	-1.184	6	0.0087	-1.447	6	0.0411	-1.213	6	0.026	-1.308
IL-18 BPa	O95998	6	0.0022	-2.311	6	0.0022	-1.813	6	0.0022	-2.585	6	0.0022	-1.736	6	0.0022	-1.935
IL-18 Rb	O95256	6	0.005	1.216	6	0.005	1.151	6	0.0303	1.264	6	0.005	1.171	6	0.005	1.243
IL-5 Ra	Q01344	6	0.0087	1.225	6	0.0087	1.2	6	0.0087	1.408	6	0.0152	1.168	6	0.0152	1.295
IL-8	P10145	6	0.0043	1.185	6	0.0087	1.17	6	0.026	1.298	6	0.0087	1.18	6	0.026	1.281
JAG1	P78504	6	0.0087	-1.342	6	0.0087	-1.293	6	0.0022	-1.75	6	ns	ns	6	0.0152	-1.386
LAG-1	Q8NHW4	6	0.0022	1.253	6	0.0022	1.274	6	0.0411	1.249	6	ns	ns	6	0.0087	1.333

Supplementary Table 1 - continued

Protein	Uniprot	n	G2 p-val	G2 FC	n	G6 p-val	G6 FC	n	G7 p-val	G7 FC	n	G8 p-val	G8 FC	n	G9 p-val	G9 FC
LEG9	O00182	6	0.0087	1.464	6	0.0087	1.248	6	0.026	1.646	6	0.026	1.211	6	ns	ns
LGMN	Q99538	6	0.0022	-1.301	6	0.0022	-1.316	6	0.0022	-1.472	6	0.0022	-1.311	6	0.0022	-1.448
LSAMP	Q13449	6	0.0022	-1.767	6	0.0043	-1.54	6	0.0022	-2.288	6	0.0043	-1.422	6	0.0087	-1.584
MATN2	O00339	6	0.0043	-1.712	6	0.0152	-1.491	6	0.0022	-2.348	6	0.0022	-1.776	6	0.0022	-1.965
MIC-1	Q99988	6	0.0081	1.28	6	0.0087	1.277	6	0.0022	1.632	6	0.0022	1.308	6	0.0022	1.524
NADPH-P450 Oxidoreductase	P16435	6	0.0087	1.226	6	0.0043	1.448	6	0.0152	1.422	6	0.0043	1.398	6	0.0043	1.56
Nectin-like protein 1	Q8N126	6	0.0087	1.511	6	0.0043	1.367	6	0.0087	1.501	6	0.0022	1.4	6	0.0411	1.624
NPS-PLA2	P14555	6	0.0022	1.333	6	0.0129	1.317	6	0.0411	1.792	6	0.0022	1.329	6	0.0087	1.56
OSM	P13725	6	0.0087	1.341	6	0.0043	1.407	6	0.0152	1.871	6	0.0043	1.385	6	0.0152	1.634
PAK7	Q9P286	6	0.0022	1.192	6	0.0022	1.222	6	0.0152	1.416	6	0.0022	1.257	6	0.0411	1.343
PCSK7	Q16549	6	0.0043	1.214	6	0.0022	1.345	6	0.0043	1.44	6	0.0022	1.312	6	0.0087	1.452
PDE11	Q9HCR9	6	0.0022	1.443	6	0.0043	1.327	6	0.0087	1.345	6	0.0043	1.251	6	0.0022	1.273
PEDF	P36955	6	0.0022	-1.648	6	0.0022	-1.425	6	0.0022	-2.025	6	0.0022	-1.395	6	0.0022	-1.61
PIM1	P11309	6	0.0022	1.442	6	0.0022	1.361	6	0.0022	1.483	6	0.0043	1.249	6	0.0022	1.353
Plasmin	P00747	6	0.0022	1.208	6	0.0022	1.247	6	0.0152	1.382	6	0.0022	1.242	6	0.026	1.299
PlGF	P49763	6	0.0022	1.77	6	0.0022	1.236	6	0.0303	1.258	6	ns	ns	6	0.0022	1.318
PRL	P01236	6	0.0022	1.408	6	0.0022	1.34	6	0.0152	1.377	6	0.0022	1.328	6	0.026	1.461
Properdin	P27918	6	0.0087	1.252	6	0.0152	1.203	6	0.0087	1.5	6	ns	ns	6	0.0152	1.283
Protease nexin I	P07093	6	0.0087	1.381	6	0.0411	1.238	6	0.0411	2.367	6	0.026	1.317	6	ns	ns

Supplementary Table 1 - continued

Protein	Uniprot	n	G2 p-val	G2 FC	n	G6 p-val	G6 FC	n	G7 p-val	G7 FC	n	G8 p-val	G8 FC	n	G9 p-val	G9 FC
Protein S	P07225	6	0.0043	-1.394	6	0.0043	-1.2	6	0.0087	-1.723	6	ns	ns	6	0.0043	-1.357
Rb	P06400	6	0.0087	1.402	6	0.0152	1.454	6	0.0152	1.849	6	0.0087	1.612	6	0.0411	1.888
RET	P07949	6	0.0043	1.272	6	0.0022	1.206	6	0.0087	1.359	6	ns	ns	6	0.0022	1.345
RGMA	Q96B86	6	0.0022	-1.586	6	0.0022	-1.365	6	0.0043	-1.94	6	0.0152	-1.32	6	0.0087	-1.47
RGM-C	Q6ZVN8	6	0.0022	-1.58	6	0.0043	-1.34	6	0.0043	-2.006	6	0.0043	-1.417	6	0.0043	-1.557
RXFP1	Q9HBX9	6	0.0064	1.258	6	0.0043	1.096	6	0.0087	1.197	6	ns	ns	6	0.0022	1.215
S100A4	P26447	6	0.0022	-1.662	6	0.0022	-1.701	6	0.0152	-1.942	6	0.0022	-1.613	6	0.0411	-1.741
sCD4	P01730	6	0.0043	1.29	6	0.0022	1.348	6	0.0087	1.451	6	0.0022	1.37	6	0.0152	1.51
Semaphorin-6A	Q9H2E6	6	0.0022	-1.746	6	0.0043	-1.472	6	0.0022	-2.212	6	0.0043	-1.473	6	0.0022	-1.829
sFRP-3	Q92765	6	0.0087	1.385	6	0.026	1.226	6	0.0411	1.763	6	0.0411	1.26	6	ns	ns
sICAM-3	P32942	6	0.0087	1.359	6	0.0022	1.316	6	0.026	1.665	6	0.0022	1.339	6	0.0152	1.525
SLAF6	Q96DU3	6	0.005	1.822	6	0.005	1.264	6	0.005	1.706	6	0.045	1.575	6	0.0081	1.551
SNP25	P60880	6	0.0087	1.618	6	0.0022	1.206	6	0.0043	1.555	6	0.0411	1.146	6	0.0043	1.415
Sphingosine kinase 1	Q9NYA1	6	0.0022	-1.654	6	0.0087	-1.512	6	0.0043	-1.803	6	0.0152	-1.42	6	0.0087	-1.632
TAFI	Q96IY4	6	0.0087	1.193	6	0.0087	1.193	6	0.0161	1.484	6	ns	ns	6	0.045	1.26
Testican-1	Q08629	6	0.0087	-1.251	6	ns	ns	6	0.0152	-1.385	6	0.026	-1.19	6	0.026	-1.299
TF	P13726	6	0.0043	-1.566	6	0.0152	-1.317	6	0.0043	-1.925	6	0.0087	-1.3	6	0.0411	-1.428
Thymidine kinase	P04183	6	0.0043	1.305	6	0.0022	1.306	6	0.0087	1.474	6	0.0022	1.308	6	0.0087	1.475
TNFSF15	O95150	6	0.0022	-1.575	6	0.0022	-1.572	6	0.0022	-1.528	6	0.0022	-1.534	6	0.0022	-1.576
TNFSF18	Q9UNG2	6	0.0087	1.199	6	0.0022	1.254	6	0.0087	1.429	6	0.0087	1.198	6	0.0152	1.272

Supplementary Table 1 - continued

Protein	Uniprot	n	G2 p-val	G2 FC	n	G6 p-val	G6 FC	n	G7 p-val	G7 FC	n	G8 p-val	G8 FC	n	G9 p-val	G9 FC
Transketolase	P29401	6	0.0022	-1.482	6	0.0152	-1.291	6	0.026	-1.907	6	ns	ns	6	0.0087	-1.568
VEGF	P15692	6	0.0087	1.184	6	0.0043	1.2	6	ns	ns	6	0.0022	1.306	6	0.0087	1.207
VEGF sR3	P35916	6	0.0022	-2.345	6	0.0022	-1.846	6	0.0022	-2.794	6	0.0022	-1.723	6	0.0043	-2.011

Supplementary Table 1a. Summary of candidate efficacy and safety biomarkers for vamorolone and prednisolone. Shown are proteins that were significant by Group 1 (vehicle) vs. Group 2 (prednisolone), and then also tested as significant in the two vamorolone validation groups comparisons (p<0.05; Group 1 vs. Groups 10, 11). ns indicates "not significant"

Protein Identifying Information	Uniprot ID	Prednisolone (Group 2)			Vamorolone (Group 10)			Vamorolone + BAIBA low dose + Rituximab Treatment Group (Group 11)		
		n	P-value (comparison to vehicle)	Fold Change(comparison to vehicle)	n	P-value (comparison to vehicle)	Fold Change(comparison to vehicle)	n	P-value (comparison to vehicle)	Fold Change(comparison to vehicle)
14-3-3 protein zeta/delta	P63104	6	0.0087	-1.392	6	ns	ns	6	ns	ns
AGR2	O95994	6	0.0081	1.206	6	0.045	1.139	6	0.005	1.244
ALCAM	Q13740	6	0.0087	-1.504	6	ns	ns	6	0.0087	-1.291
Alkaline phosphatase, bone	P05186	6	0.0022	-2.811	6	ns	ns	6	ns	ns
Apo A-I	P02647	6	0.0087	1.44	6	0.0129	1.253	6	0.0043	1.45
ARSB	P15848	6	0.0087	1.191	6	0.0411	1.112	6	0.026	1.187
AURKB	Q96GD4	6	0.0087	1.221	6	0.0411	1.115	6	0.0087	1.145
Aurora kinase A	O14965	6	0.0087	1.592	6	ns	ns	6	0.0087	1.371
B7-H2	O75144	6	0.0022	-1.173	6	ns	ns	6	ns	ns
BCL2-like 1 protein	Q07817	6	0.0043	1.288	6	0.0043	1.183	6	0.0022	1.297
bFGF-R	P11362	6	0.0022	-1.568	6	ns	ns	6	0.0043	-1.312
BGH3	Q15582	6	0.0087	-1.564	6	ns	ns	6	0.0022	-1.632
C34 gp41 HIV Fragment	Q70626	6	0.0022	1.671	6	ns	ns	6	ns	ns
Cadherin-5	P33151	6	0.0022	-2.122	6	0.0087	-1.286	6	0.0022	-1.643
CATE	P14091	6	0.0087	-1.408	6	ns	ns	6	0.0152	-1.272
CATF	Q9UBX1	6	0.0043	-2.782	6	0.0022	-2.682	6	ns	ns

Supplementary Table 1a - continued

Protein	Uniprot	n	G2 p-val	G2 FC	n	G10 p-val	G10 FC	n	G11 p-val	G11 FC
Cathepsin A	P10619	6	0.0087	1.305	6	0.026	1.152	6	0.026	1.798
CD23	P06734	6	0.0022	1.377	6	0.0022	1.307	6	0.0022	1.435
CD30	P28908	6	0.0022	1.335	6	ns	ns	6	0.0087	1.123
CD30 Ligand	P32971	6	0.0087	1.272	6	0.0087	1.25	6	0.0022	1.452
CD39	P49961	6	0.0087	1.24	6	0.0043	1.298	6	0.0022	1.376
CD83	Q01151	6	0.0022	-1.274	6	ns	ns	6	ns	ns
CDK2/cyclin A	P24941 P20248	6	0.0022	-1.677	6	0.0022	-1.498	6	0.0022	-1.672
Chitotriosidase-1	Q13231	6	0.0087	1.377	6	0.0087	1.319	6	0.0022	1.395
Chymase	P23946	6	0.0043	1.447	6	ns	ns	6	0.0087	1.271
Ck-b-8-1	P55773	6	0.0022	1.227	6	0.0043	1.225	6	0.0022	1.186
CK-MB	P12277 P06732	6	0.0043	-2.402	6	0.026	-1.734	6	0.0087	-2.125
c-Myc	P01106	6	0.0043	1.498	6	ns	ns	6	0.0247	1.115
DLL1	O00548	6	0.0022	-1.789	6	0.0087	-1.273	6	0.0022	-1.556
DRAK2	O94768	6	0.0087	1.43	6	0.026	1.33	6	0.0043	1.434
Dtk	Q06418	6	0.0043	1.204	6	0.0043	1.329	6	0.0022	1.388
EGF	P01133	6	0.0087	1.656	6	0.0087	1.241	6	0.0022	1.604
Eotaxin-2	O00175	6	0.0022	1.329	6	0.0043	1.136	6	0.0022	1.219
EPHB2	P29323	6	0.0022	-1.387	6	ns	ns	6	0.0043	-1.221
EphB6	O15197	6	0.0087	-1.46	6	ns	ns	6	0.026	-1.236
FGF-17	O60258	6	0.0049	1.111	6	0.005	1.146	6	0.0049	1.165
FLRT3	Q9NZU0	6	0.0087	-1.374	6	ns	ns	6	0.0087	-1.389
FSTL1	Q12841	6	0.0087	-1.556	6	ns	ns	6	0.026	-1.559
GFRa-2	O00451	6	0.0022	-1.578	6	0.0411	-1.163	6	0.0022	-1.395
GITR	Q9Y5U5	6	0.0022	-1.691	6	ns	ns	6	0.0087	-1.323
Glypican 3	P51654	6	0.0087	1.882	6	ns	ns	6	ns	ns

Supplementary Table 1a - continued

Protein	Uniprot	n	G2 p-val	G2 FC	n	G10 p-val	G10 FC	n	G11 p-val	G11 FC
HAI-1	O43278	6	0.0043	1.258	6	0.0043	1.317	6	0.0022	1.391
HSP 90b	P08238	6	0.0087	-1.53	6	ns	ns	6	ns	ns
HXK1	P19367	6	0.0087	1.706	6	ns	ns	6	0.0043	1.333
I-309	P22362	6	0.0043	1.21	6	0.0043	1.082	6	0.0152	1.13
IGFBP-5	P24593	6	0.0087	-1.424	6	0.0152	-1.273	6	0.0022	-1.434
IL-12 RB2	Q99665	6	0.0087	-1.275	6	ns	ns	6	ns	ns
IL-18 BPa	O95998	6	0.0022	-2.311	6	0.0087	-1.215	6	0.0022	-1.527
IL-18 Rb	O95256	6	0.005	1.216	6	0.005	1.138	6	0.005	1.24
IL-5 Ra	Q01344	6	0.0087	1.225	6	0.026	1.161	6	0.0081	1.215
IL-8	P10145	6	0.0043	1.185	6	0.0152	1.166	6	0.0022	1.383
JAG1	P78504	6	0.0087	-1.342	6	ns	ns	6	0.0087	-1.303
LAG-1	Q8NHW4	6	0.0022	1.253	6	0.0043	1.253	6	0.005	1.317
LEG9	O00182	6	0.0087	1.464	6	ns	ns	6	0.0087	1.258
LGMN	Q99538	6	0.0022	-1.301	6	0.026	-1.228	6	0.0087	-1.386
LSAMP	Q13449	6	0.0022	-1.767	6	ns	ns	6	0.0022	-1.397
MATN2	O00339	6	0.0043	-1.712	6	0.0087	-1.466	6	0.0022	-1.844
MIC-1	Q99988	6	0.0081	1.28	6	0.0152	1.189	6	0.0022	1.322
NADPH-P450 Oxidoreductase	P16435	6	0.0087	1.226	6	0.0087	1.314	6	0.0043	1.496
Nectin-like protein 1	Q8N126	6	0.0087	1.511	6	0.0043	1.409	6	0.0043	1.478
NPS-PLA2	P14555	6	0.0022	1.333	6	0.0103	1.313	6	0.0022	61.215
OSM	P13725	6	0.0087	1.341	6	0.0152	1.29	6	0.0022	1.432
PAK7	Q9P286	6	0.0022	1.192	6	0.0087	1.215	6	0.0022	1.292
PCSK7	Q16549	6	0.0043	1.214	6	0.0087	1.249	6	0.0022	1.344
PDE11	Q9HCR9	6	0.0022	1.443	6	ns	ns	6	ns	ns
PEDF	P36955	6	0.0022	-1.648	6	ns	ns	6	0.0152	-1.235
PIM1	P11309	6	0.0022	1.442	6	0.026	1.168	6	0.0043	1.174

Supplementary Table 1a - continued

Protein	Uniprot	n	G2 p-val	G2 FC	n	G10 p-val	G10 FC	n	G11 p-val	G11 FC
Plasmin	P00747	6	0.0022	1.208	6	0.0087	1.195	6	0.0081	1.228
PlGF	P49763	6	0.0022	1.77	6	0.0411	1.073	6	0.0411	1.14
PRL	P01236	6	0.0022	1.408	6	0.0087	1.345	6	0.0087	1.319
Properdin	P27918	6	0.0087	1.252	6	0.026	1.2	6	0.0022	1.373
Protease nexin I	P07093	6	0.0087	1.381	6	ns	ns	6	0.0022	1.478
Protein S	P07225	6	0.0043	-1.394	6	ns	ns	6	0.0022	-1.258
Rb	P06400	6	0.0087	1.402	6	0.0152	1.386	6	0.0022	1.568
RET	P07949	6	0.0043	1.272	6	0.0081	1.16	6	0.0022	1.203
RGMA	Q96B86	6	0.0022	-1.586	6	ns	ns	6	0.0022	-1.298
RGM-C	Q6ZVN8	6	0.0022	-1.58	6	ns	ns	6	ns	ns
RXFP1	Q9HBX9	6	0.0064	1.258	6	0.0087	1.093	6	0.0087	1.08
S100A4	P26447	6	0.0022	-1.662	6	0.0022	-1.588	6	0.026	-1.647
sCD4	P01730	6	0.0043	1.29	6	0.0022	1.352	6	0.0022	1.5
Semaphorin-6A	Q9H2E6	6	0.0022	-1.746	6	0.0043	-1.321	6	0.0022	-1.49
sFRP-3	Q92765	6	0.0087	1.385	6	0.026	1.188	6	0.0087	1.322
sICAM-3	P32942	6	0.0087	1.359	6	0.0087	1.292	6	0.0022	1.324
SLAF6	Q96DU3	6	0.005	1.822	6	0.0081	1.2	6	0.005	1.313
SNP25	P60880	6	0.0087	1.618	6	ns	ns	6	0.0087	1.17
Sphingosine kinase 1	Q9NYA1	6	0.0022	-1.654	6	ns	ns	6	0.0152	-1.287
TAFI	Q96IY4	6	0.0087	1.193	6	0.0043	1.163	6	0.0022	1.24
Testican-1	Q08629	6	0.0087	-1.251	6	0.026	-1.15	6	0.0022	-1.211
TF	P13726	6	0.0043	-1.566	6	ns	ns	6	0.0411	-1.253
Thymidine kinase	P04183	6	0.0043	1.305	6	0.0022	1.262	6	0.0022	1.368
TNFSF15	O95150	6	0.0022	-1.575	6	0.0022	-1.383	6	0.0022	-1.371
TNFSF18	Q9UNG2	6	0.0087	1.199	6	0.0087	1.141	6	0.0022	1.228

Supplementary Table 1a - continued

Protein	Uniprot	n	G2 p-val	G2 FC	n	G10 p-val	G10 FC	n	G11 p-val	G11 FC
Transketolase	P29401	6	0.0022	-1.482	6	ns	ns	6	0.0043	-1.538
VEGF	P15692	6	0.0087	1.184	6	0.0152	1.16	6	0.0152	1.142
VEGF sR3	P35916	6	0.0022	-2.345	6	ns	ns	6	0.0022	-1.556

Supplementary Table 2. Results of this study applied to the list of significant proteins found by Hathout et al. 2016. Gold and pale yellow, results below 100±10%, Green, protein is down regulated in humans, Red, protein is upregulated in humans.

Human Glucocorticoid Responsive Biomarker	UniProt ID	Vehicle	Prednisolone		Rituximab + prednisolone		BIABA low dose + prednisolone		BIABA high dose + prednisolone		BIABA low dose + Rituximab + prednisolone		Vamorolone		Vamorolone + BAIBA low dose + Rituximab treatment group	
		Mean ± SD	Mean ± SD	P value vs. vehicle	Mean ± SD	P value vs. vehicle	Mean ± SD	P value vs. vehicle	Mean ± SD	P value vs. vehicle	Mean ± SD	P value vs. vehicle	Mean ± SD	P value vs. vehicle	Mean ± SD	P value vs. vehicle
6Ckine	O00585	213.6 ± 22.9	190.9 ± 19.5	0.132	201.2 ± 10.7	0.485	201.2 ± 23.1	0.485	214.9 ± 21	0.818	213.5 ± 34.9	0.937	209.1 ± 17.1	0.699	210 ± 13.8	0.937
a1-Antitrypsin	P01009	2940.9 ± 233.8	2683 ± 676.5	0.818	2807 ± 248.2	0.485	3520.4 ± 535.1	0.065	2937 ± 330.7	0.818	2763.2 ± 265.6	0.31	2967.1 ± 115.6	0.699	2933.8 ± 137.7	1
Afamin	P43652	253.5 ± 60.1	393.5 ± 93.4	0.026	394.6 ± 87.6	0.009	679.5 ± 366.8	0.026	410.1 ± 119.2	0.015	509.3 ± 289.2	0.065	374.8 ± 74.4	0.015	471.9 ± 93.6	0.002
Angiopoietin-2	O15123	61.3 ± 20.2	52.7 ± 17.8	0.31	50.8 ± 6.4	0.078	36.5 ± 17.7	0.065	44 ± 12.6	0.132	47.1 ± 12.8	0.24	55.1 ± 4.7	0.132	49.1 ± 11.6	0.24
Angiotensinogen	P01019	102.4 ± 70.2	143.2 ± 45.2	0.24	129.8 ± 67.3	0.485	119.6 ± 56.3	0.937	127 ± 68.4	0.699	122.5 ± 58	0.24	88 ± 51.6	0.818	142.7 ± 66.9	0.485
C1r	P00736	28.4 ± 4.5	28.1 ± 6.3	1	26.5 ± 3.5	0.394	21.3 ± 7.6	0.093	28.7 ± 4.7	0.937	25.4 ± 7.1	0.485	28.5 ± 2.3	0.937	23.1 ± 4.1	0.065
CD23	P06734	162.4 ± 20.9	223.6 ± 37.6	0.002	219.4 ± 32.9	0.002	281.9 ± 86.1	0.026	219.4 ± 34	0.004	266.6 ± 102.6	0.026	212.3 ± 26	0.002	233.1 ± 33.9	0.002
CD36 ANTIGEN	P16671	267.8 ± 15.9	279.6 ± 18.4	0.24	288.6 ± 7.2	0.045	379.4 ± 212.2	0.041	294.2 ± 19.6	0.026	372.4 ± 227.2	0.093	278.6 ± 3.6	0.132	301.6 ± 10.6	0.004
CNDP1	Q96KN2	209.5 ± 109.5	252.5 ± 130.1	0.336	188.4 ± 90.4	1	290.1 ± 106.2	0.18	211.6 ± 99.3	0.937	190.1 ± 77.2	0.937	163.6 ± 32.7	0.818	280.1 ± 114	0.31

Supplementary Table 2 - continued

Protein	Uniprot	G1 mean	G2 mean	G2 p-value	G6 mean	G6 p-value	G7 mean	G7 p-value	G8 mean	G8 p-value	G9 mean	G9 p-value	G10 mean	G10 p-value	G11 mean	G11 p-value
CNTF	P26441	50 ± 7.8	53.9 ± 18.7	0.699	52.6 ± 11.7	0.937	43.7 ± 7	0.18	43.6 ± 7.2	0.18	46.8 ± 9.8	0.485	49.2 ± 5.5	0.63	45.1 ± 3.9	0.199
CRDL1	Q9BU40	2375 ± 349.2	2216.6 ± 592.6	0.818	2471.8 ± 525.7	0.589	1708.4 ± 559	0.065	2305.6 ± 434.8	0.31	1942.5 ± 469.7	0.18	2222.8 ± 421.7	0.485	1998.3 ± 444.1	0.132
EMR2	Q9UHX3	68.6 ± 10	64.8 ± 10.8	0.485	68 ± 5.2	1	55.6 ± 15.3	0.132	61.7 ± 15.3	0.485	69.8 ± 9.8	1	69.2 ± 8.4	0.937	63.1 ± 5.2	0.422
Fibrinogen g-chain dimer	P02679	355862.8 ± 186762.6	136884.3 ± 83592.8	0.065	211668.8 ± 161525.5	0.18	122268.9 ± 185658.8	0.026	194675.3 ± 174861.1	0.093	180849.1 ± 204335	0.132	283244.1 ± 200232.4	0.699	133647 ± 113567.2	0.041
Growth hormone receptor	P10912	70.4 ± 8.6	98.7 ± 57.2	0.31	76.8 ± 6.1	0.336	94.5 ± 21.6	0.065	77.2 ± 5	0.18	85.4 ± 14.3	0.041	73.2 ± 2.5	0.937	78.7 ± 7.3	0.132
IFN-aA	P01563	50.5 ± 2.9	44.7 ± 4.7	0.026	48.5 ± 2.4	0.18	43.2 ± 5.1	0.054	47.1 ± 11	0.699	48.3 ± 4.9	0.18	48.6 ± 2.7	0.394	44.3 ± 1.7	0.002
IGFBP-1	P08833	6498.2 ± 5856.6	6813.4 ± 11483.7	0.394	9229.5 ± 8950.7	0.937	7903.3 ± 11823.5	0.589	9835.4 ± 18269.7	0.485	9577.9 ± 10472.2	0.937	8410.1 ± 8158.1	0.699	6952.6 ± 6554	0.818
IGFBP-2	P18065	75479.5 ± 35786.8	41044.7 ± 19228.3	0.132	46423.2 ± 15079.7	0.132	45864 ± 31101.3	0.24	61239.9 ± 33214.6	0.31	50990.1 ± 14064.2	0.132	104005.9 ± 30129.4	0.132	78348.9 ± 21952.5	0.699
IgM	P01871	107.7 ± 9.7	159.7 ± 88.3	0.093	113.4 ± 4.8	0.18	138.1 ± 26.1	0.015	335.3 ± 364.6	0.18	134.3 ± 39.8	0.065	104 ± 7.1	0.699	114.3 ± 10	0.24
IL-22BP	Q969J5	140.8 ± 9.1	143.9 ± 36.6	0.24	132.9 ± 3.6	0.093	125.2 ± 10.4	0.026	140.4 ± 7.8	0.937	140.2 ± 14.9	0.699	134.7 ± 5.7	0.18	138.6 ± 5.1	0.589
Insulin	P01308	60.4 ± 10.1	56.4 ± 9.1	0.394	58 ± 6.2	0.485	42.7 ± 18.9	0.132	64.1 ± 19.3	0.937	58.1 ± 16.3	0.589	55.5 ± 8.2	0.24	121.2 ± 169.7	0.31

Supplementary Table 2 - continued

Protein	Uniprot	G1 mean	G2 mean	G2 p-value	G6 mean	G6 p-value	G7 mean	G7 p-value	G8 mean	G8 p-value	G9 mean	G9 p-value	G10 mean	G10 p-value	G11 mean	G11 p-value
Integrin a1b1	P56199, P05556	19102.8 ± 11950.5	13965.2 ± 7411.5	0.485	12266.8 ± 4502.2	0.31	13771.5 ± 4876.4	0.485	12889 ± 6162	0.24	11984.5 ± 4706.7	0.093	17062.4 ± 10780	0.485	18197.1 ± 14346	0.818
Leptin	P41159	453.7 ± 30.6	479.1 ± 65.8	0.818	431.4 ± 24.4	0.24	437.6 ± 37	0.818	444.4 ± 75.8	0.24	423.8 ± 31.9	0.24	406.8 ± 31.6	0.041	438.5 ± 36.3	0.589
LY9	Q9HBG7	65.2 ± 36.3	65.1 ± 22.6	0.485	73.8 ± 35.7	0.199	43.3 ± 17.6	0.18	82.1 ± 77.7	0.818	55.9 ± 11.6	1	57.6 ± 6.5	0.575	49.5 ± 5.8	0.699
Lymphotoxin a2/b1	P01374, Q06643	355.2 ± 29	388.5 ± 57.9	0.18	386.8 ± 34.1	0.132	509.6 ± 128	0.065	358.8 ± 60.6	0.589	375 ± 37.2	0.31	362.4 ± 25.1	0.589	372.4 ± 23.4	0.394
M-CSF R	P07333	56.4 ± 8.1	57 ± 18.3	0.485	55.4 ± 5.5	0.699	49.2 ± 8.5	0.24	54.9 ± 10.9	0.485	51.7 ± 11.8	0.485	55.5 ± 4.3	0.485	47.3 ± 3	0.065
MDC	O00626	113.2 ± 7.5	114.2 ± 9.1	0.937	116.8 ± 5.9	0.394	111 ± 10.5	0.937	115.6 ± 11.3	0.229	119.8 ± 7.9	0.31	116.2 ± 9.1	0.937	141.7 ± 62.6	0.18
MMP-12	P39900	48.9 ± 8.1	47 ± 10.4	0.937	43.6 ± 4.9	0.394	34.1 ± 14.2	0.093	43.6 ± 8.9	0.422	42 ± 13	0.31	45.9 ± 6.7	0.262	39.6 ± 6.8	0.065
MMP-3	P08254	163.8 ± 19.7	185 ± 22.8	0.18	182.9 ± 16.1	0.132	213.3 ± 51.3	0.132	194.1 ± 28.8	0.065	196.8 ± 30.9	0.109	185.6 ± 16.2	0.065	207.3 ± 26.8	0.026
MPIF-1	P55773	185.6 ± 14.6	199.8 ± 12.4	0.132	219.3 ± 19.4	0.004	220.5 ± 28.8	0.025	215.4 ± 23.5	0.015	243.2 ± 35.6	0.002	207.6 ± 12	0.015	213 ± 16.7	0.015
NCAM-120	P13591	162.8 ± 45.2	194.2 ± 40.6	0.31	126.8 ± 27.6	0.18	264.6 ± 196.6	0.485	160.8 ± 93	0.589	128.1 ± 41.9	0.31	141.4 ± 45.9	0.18	223.6 ± 97.6	0.31
P-Cadherin	P22223	5757.7 ± 881.4	5128.4 ± 783.7	0.31	5538.8 ± 630	1	4529.2 ± 1432.9	0.18	5464.9 ± 1076.1	0.485	4988.4 ± 1107.5	0.31	5350.6 ± 494.5	0.485	6234.1 ± 3581.5	0.18
Protein C	P04070	335.6 ± 57.6	415.1 ± 126.1	0.485	401 ± 96	0.394	773.2 ± 348.5	0.026	430.1 ± 107.9	0.18	479.6 ± 216.3	0.24	433.9 ± 164.8	0.18	503.8 ± 110.6	0.015

Supplementary Table 2 - continued

Protein	Uniprot	G1 mean	G2 mean	G2 p-value	G6 mean	G6 p-value	G7 mean	G7 p-value	G8 mean	G8 p-value	G9 mean	G9 p-value	G10 mean	G10 p-value	G11 mean	G11 p-value
Protein disulfide-isomerase	P07237	99.3 ± 14.7	82.1 ± 16.3	0.132	90 ± 9.7	0.31	75.9 ± 17.4	0.041	81.3 ± 18.4	0.093	83 ± 18.9	0.18	94.8 ± 11.2	0.394	88.1 ± 13.4	0.31
Resistin	Q9HD89	194.8 ± 12.8	328.7 ± 162.4	0.015	226.2 ± 40.4	0.093	280.1 ± 76.7	0.002	267.5 ± 128.7	0.065	322.6 ± 192.2	0.026	195.8 ± 7.1	0.937	309.1 ± 135.8	0.009
SLIK5	O94991	380.7 ± 43	268.3 ± 53.1	0.004	318.9 ± 57.3	0.065	225.8 ± 111.7	0.015	300.7 ± 65.7	0.041	297.4 ± 99.5	0.132	349.7 ± 41.3	0.31	300.5 ± 37.1	0.009
TECK	O15444	85.2 ± 7.1	99.4 ± 14	0.045	100.5 ± 6.5	0.015	95.3 ± 11.1	0.093	96.2 ± 9.7	0.041	102.1 ± 10.9	0.015	99.4 ± 9.3	0.015	98.9 ± 4.6	0.009
Testican-2	Q92563	604.5 ± 27.1	895.7 ± 162.8	0.002	1003.4 ± 164.6	0.002	821.4 ± 408.3	0.699	863.7 ± 199	0.004	740.2 ± 378.6	1	568.8 ± 110.3	0.394	550.4 ± 142.3	0.24

Supplementary Table 3. Summary of candidate efficacy and safety biomarkers for BAIBA low dose. Shown are proteins that were significant by Group 1 (vehicle) vs. Group 4 (BAIBA low dose), and then also tested as significant in all three of the validation group comparisons ($p<0.05$; Group 4 vs. Groups 7, 9, or 11).

Protein Identifying Information	Uniprot ID	BAIBA low dose (Group 4)			BAIBA low dose + Prednisolone Treatment Group (Group 7)			BAIBA low dose + Rituximab + Prednisolone Group (Group 9)			Vamorolone + BAIBA low dose + Rituximab Treatment Group (Group 11)		
		n	P-value (comparison to vehicle)	Fold Change(comparison to vehicle)	n	P-value (comparison to vehicle)	Fold Change(comparison to vehicle)	n	P-value (comparison to vehicle)	Fold Change(comparison to vehicle)	n	P-value (comparison to vehicle)	Fold Change(comparison to vehicle)
p53	P04637	6	0.0022	1.27	6	0.0411	1.654	6	0.0411	1.345	6	0.0022	1.223
S100A4	P26447	6	0.0043	-1.493	6	0.0152	-1.942	6	0.0411	-1.741	6	0.026	-1.647
Prothrombin	P00734	6	0.0043	-1.352	6	0.026	-1.716	6	0.026	-1.294	6	0.0043	-1.285
SLAF6	Q96DU3	6	0.005	1.325	6	0.005	1.706	6	0.0081	1.551	6	0.005	1.313
Protein S	P07225	6	0.0087	-1.277	6	0.0087	-1.723	6	0.0043	-1.357	6	0.0022	-1.258
IL-13	P35225	6	0.0087	1.274	6	0.0411	1.605	6	0.0129	1.659	6	0.0043	1.316
CD30 Ligand	P32971	6	0.0087	1.283	6	0.0411	1.62	6	0.026	1.467	6	0.0022	1.452
HIPK3	Q9H422	6	0.0087	1.181	6	0.0152	1.181	6	0.0087	1.396	6	0.0152	1.254
IL-8	P10145	6	0.0087	1.148	6	0.026	1.298	6	0.026	1.281	6	0.0022	1.383
Transketolase	P29401	6	0.0087	-1.383	6	0.026	-1.907	6	0.0087	-1.568	6	0.0043	-1.538

Supplementary Table 4. Summary of candidate efficacy and safety biomarkers for BAIBA high dose. Shown are proteins that were significant by Group 1 (vehicle) vs. Group 5 (BAIBA low dose), and then also tested as significant in the validation group comparison (p<0.05; Group 5 vs. Groups 8).

Protein Identifying Information	Uniprot ID	BAIBA high Treatment Group (Group 5)			BAIBA high dose + Prednisolone Treatment Group (Group 8)		
		n	P-value (comparison to vehicle)	Fold Change(comparison to vehicle)	n	P-value (comparison to vehicle)	Fold Change(comparison to vehicle)
Cathepsin G	P08311	5	0.0043	1.237	6	0.0022	1.215
Ephrin-B3	Q15768	5	0.0043	1.235	6	0.0043	1.218
PRL	P01236	5	0.0043	1.35	6	0.0022	1.328
BCMA	Q02223	5	0.0043	1.245	6	0.0087	1.249
Ck-b-8-1	P55773	5	0.0043	2.773	6	0.0087	2.639
sCD4	P01730	5	0.0043	1.344	6	0.0022	1.37
IL-2 sRa	P01589	5	0.0043	1.384	6	0.0087	1.309
CD39	P49961	5	0.0043	1.292	6	0.0022	1.372
CD109	Q6YHK3	5	0.0043	1.377	6	0.0043	1.34
CYTF	O76096	5	0.0043	1.13	6	0.0411	1.149
FCG2B	P31994	5	0.0043	1.214	6	0.0411	1.211
Chitotriosidase-1	Q13231	5	0.0043	1.399	6	0.0022	1.356
Plasmin	P00747	5	0.0043	1.257	6	0.0022	1.242
Thymidine kinase	P04183	5	0.0043	1.328	6	0.0022	1.308
PCSK7	Q16549	5	0.0043	1.34	6	0.0022	1.312
EGF	P01133	5	0.0043	1.335	6	0.0087	1.307
INGR2	P38484	5	0.0043	1.249	6	0.026	1.334
IL-18 Rb	O95256	5	0.008	1.147	6	0.005	1.171
SLAF6	Q96DU3	5	0.008	1.486	6	0.045	1.575
FGF-8B	P55075	5	0.0087	1.458	6	0.0087	1.486
Dtk	Q06418	5	0.0087	1.251	6	0.0043	1.349

Supplementary Table 4 – continued

Protein	Uniprot	n	G5 p-value	G5 FC	n	G8 p-value	G8 FC
NADPH-P450 Oxidoreductase	P16435	5	0.0087	1.368	6	0.0043	1.398
IL-17F	Q96PD4	5	0.0087	1.383	6	0.0411	1.372
HAI-1	O43278	5	0.0087	1.307	6	0.0022	1.344
Midkine	P21741	5	0.0087	1.436	6	0.0087	1.466
ER	P03372	5	0.0087	1.328	6	0.0087	1.395
NET4	Q9HB63	5	0.0087	1.212	6	0.0152	1.277
IL-15 Ra	Q13261	5	0.0087	1.353	6	0.0043	1.343
Discoidin domain receptor 2	Q16832	5	0.0087	-1.123	6	0.0152	-1.112
MMEL2	Q495T6	5	0.0087	1.379	6	0.0022	1.406
Nectin-like protein 1	Q8N126	5	0.0087	1.352	6	0.0022	1.4
ADAM 9	Q13443	5	0.0087	1.539	6	0.0152	1.629
MIC-1	Q99988	5	0.0087	1.285	6	0.0022	1.308
BCL2-like 1 protein	Q07817	5	0.0087	1.303	6	0.026	1.185
Rb	P06400	5	0.0087	1.697	6	0.0087	1.612

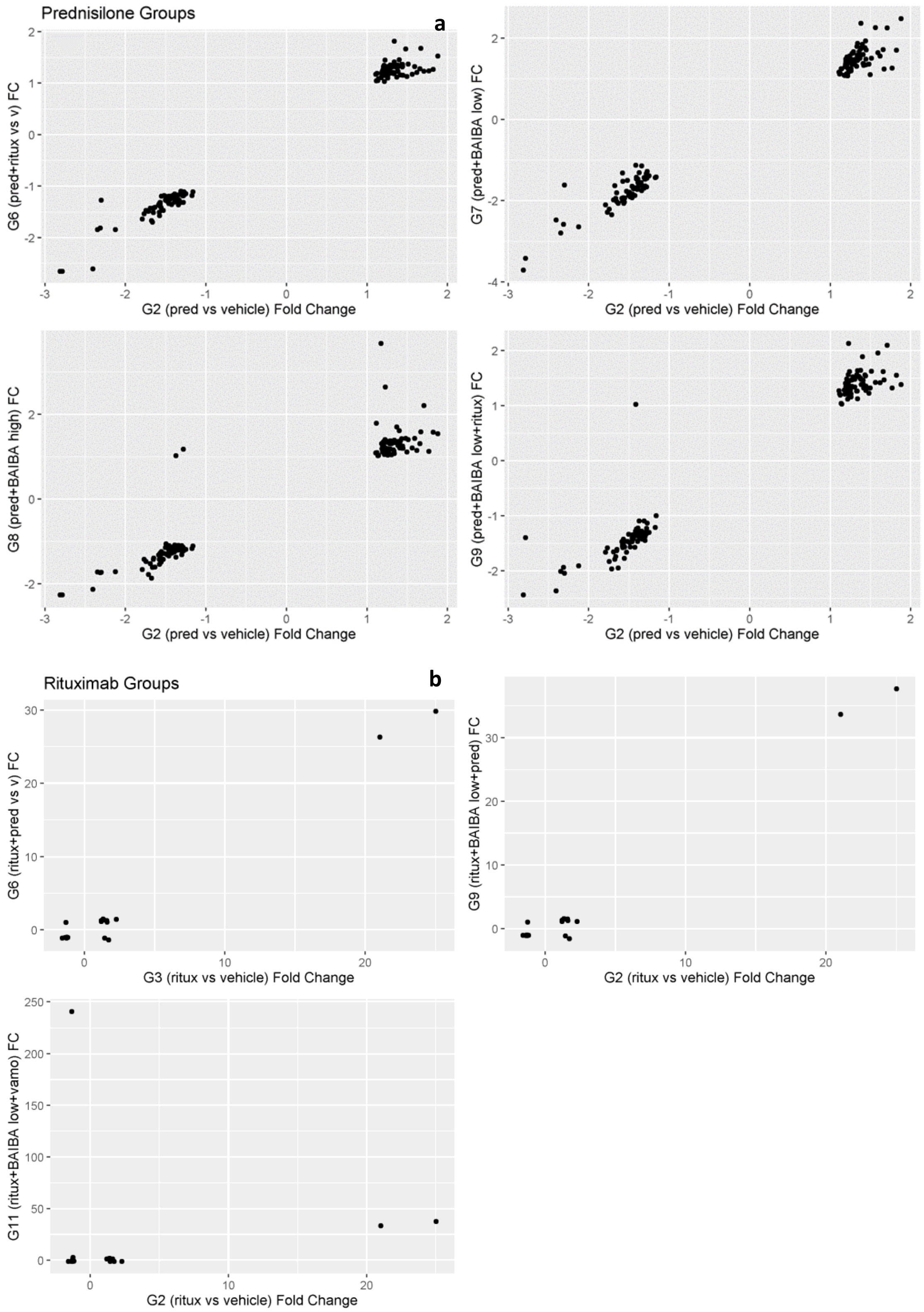

Supplementary Figure 1: Fold Change Scatter plots of fold change values for prednisolone and rituximab from drug groups (individual vs. combination). Rituximab plots seem compressed to the lower left due to large outliers.

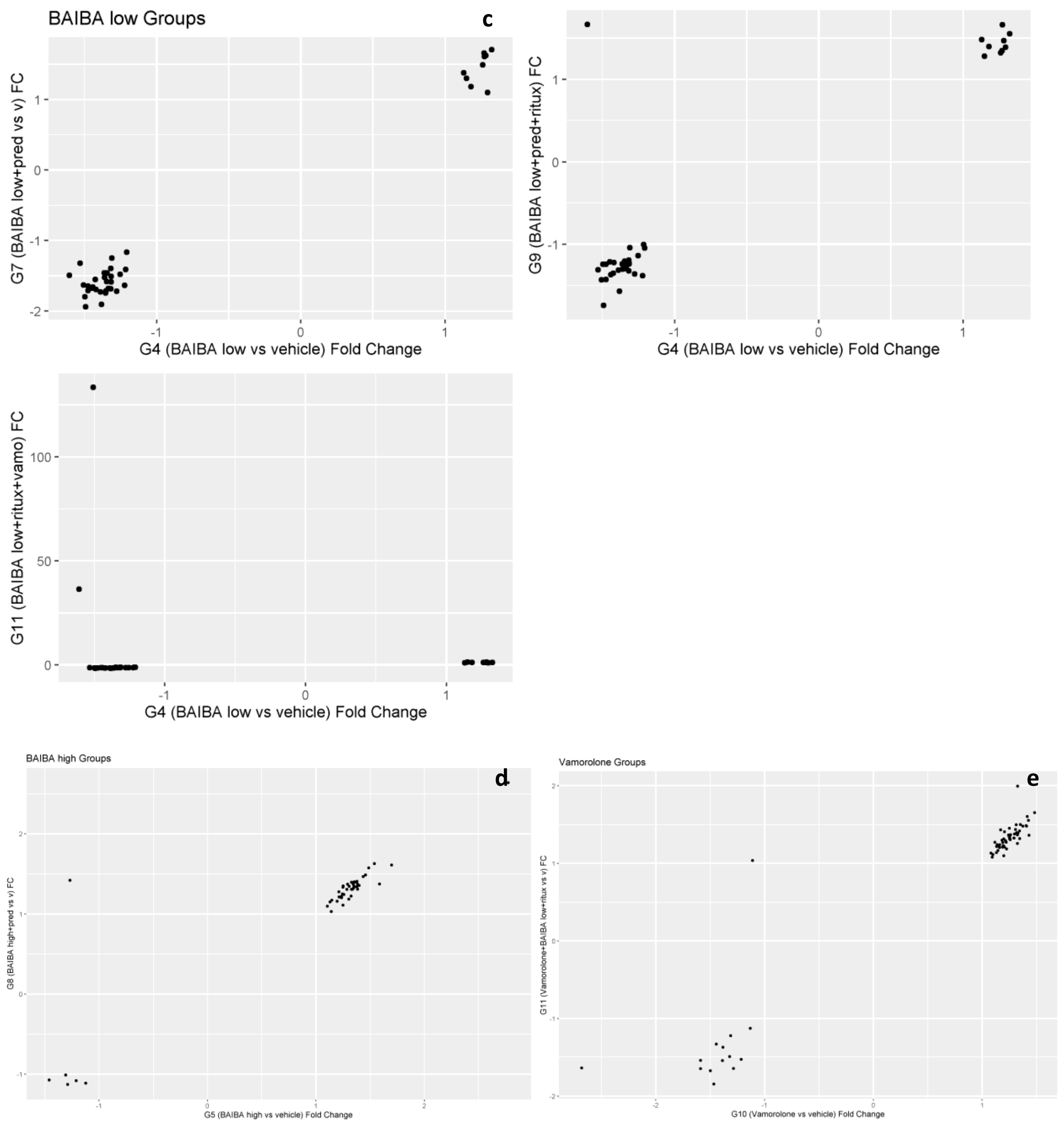

Supplementary Figure 1 continued: Fold Change Scatter plots of fold change values for prednisolone and rituximab from drug groups (individual vs. combination). BAIBA low plots seem compressed to the lower left due to large outliers.

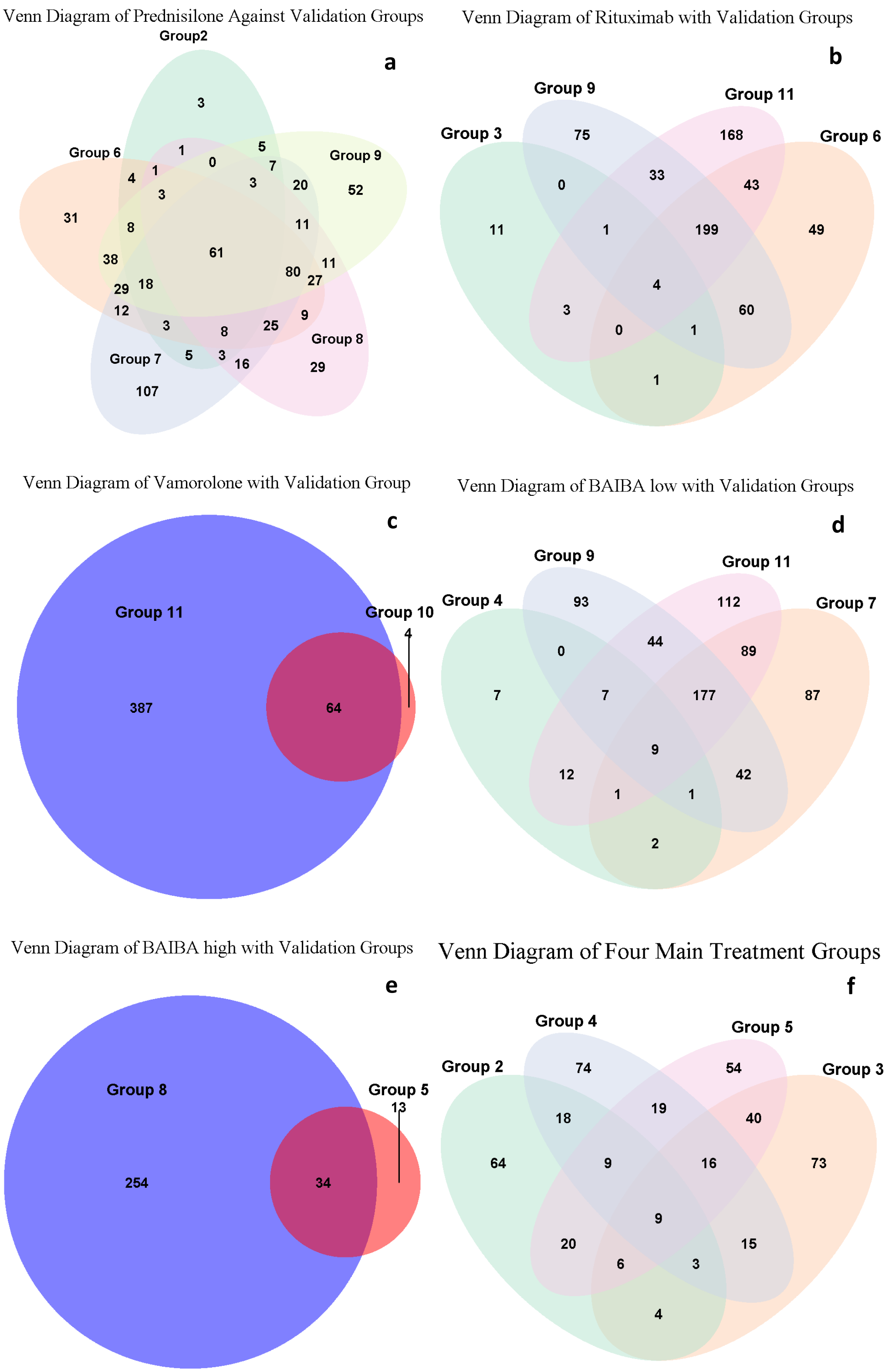

Supplementary Figure 2: Venn diagrams for significant proteins in all treatment groups. Intersections indicate commonality between groups. Specific protein details for diagrams in: Venn diagram supporting data pdf files.

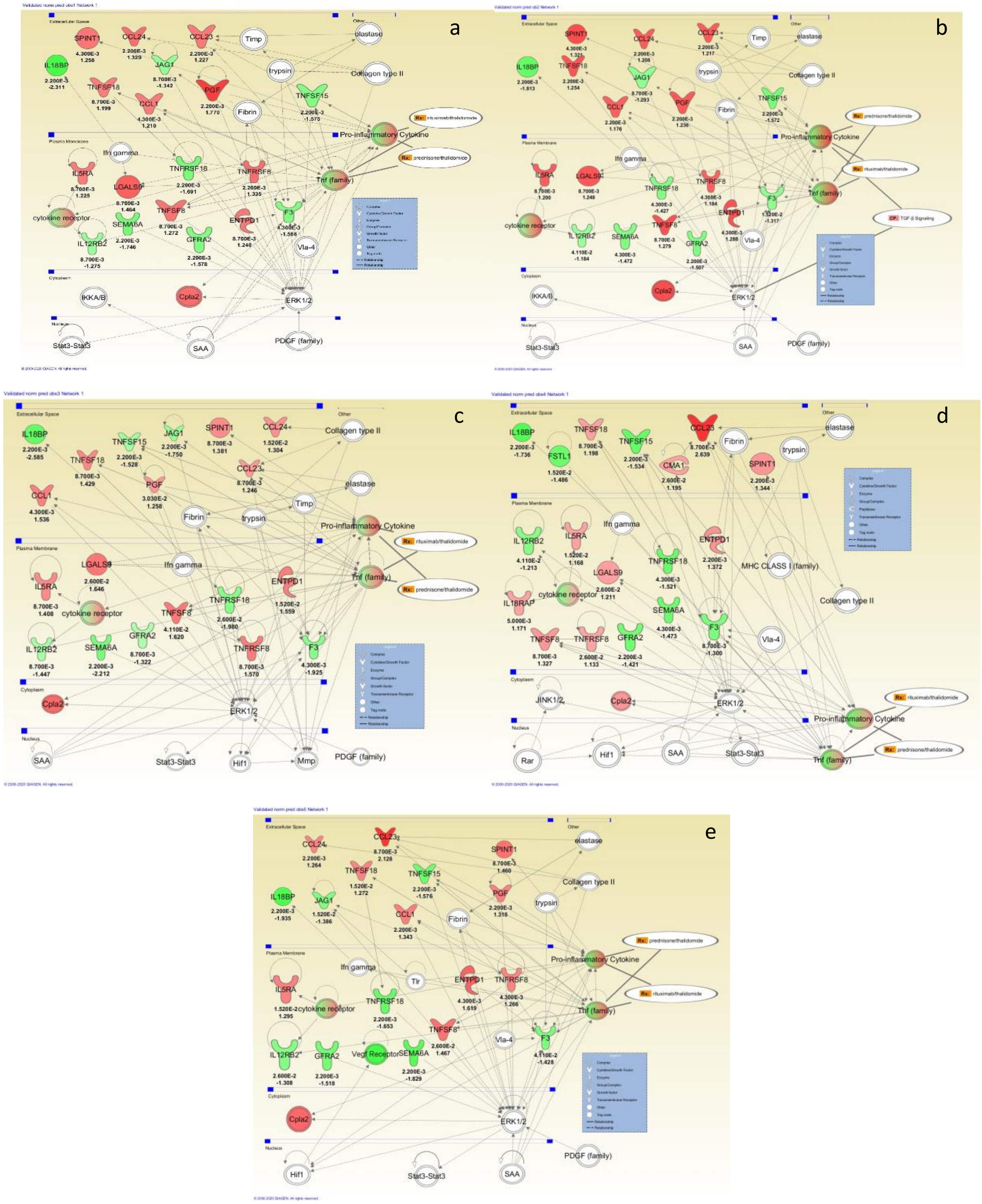

Supplementary Figure 3: IPA Diagrams for Prednisilone. Figures: a) Prednisilone only, b) Prednisilone and Rituximab, c) Prednisilone plus BAIBA low dose, d) Prednisilone plus BAIBA high dose, e) Prednisilone plus Rituximab plus BAIBA low dose. Red indicates upregulation and green indicates downregulation and color intensity correlates with size of expression change. The multicolored elements are protein complexes where the underlying data and documentation are inconsistent with IPAs search algorithms and therefore, the details are not shown.

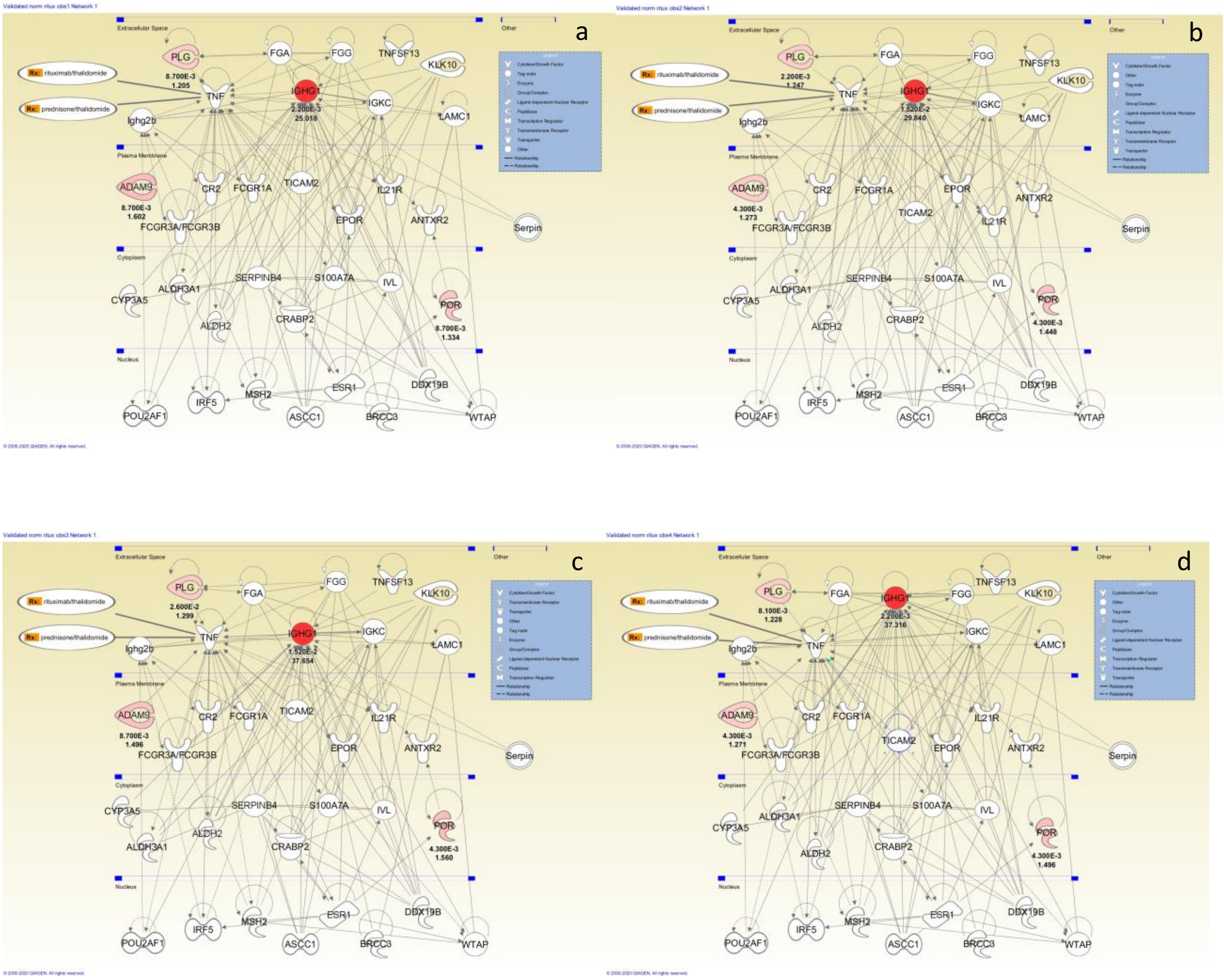

Supplementary Figure 4: IPA Diagrams for Rituximab. Figures: a) Rituximab b) Rituximab plus prednisolone c) Rituximab plus prednisilone plus BAIBA low dose, d) Rituximab plus vamorolone plus BAIBA low dose.

Red indicates upregulation and green indicates downregulation and color intensity correlates with size of expression change. The multicolored elements are protein complexes where the underlying data and documentation are inconsistent with IPAs search algorithms and therefore, the details are not shown.

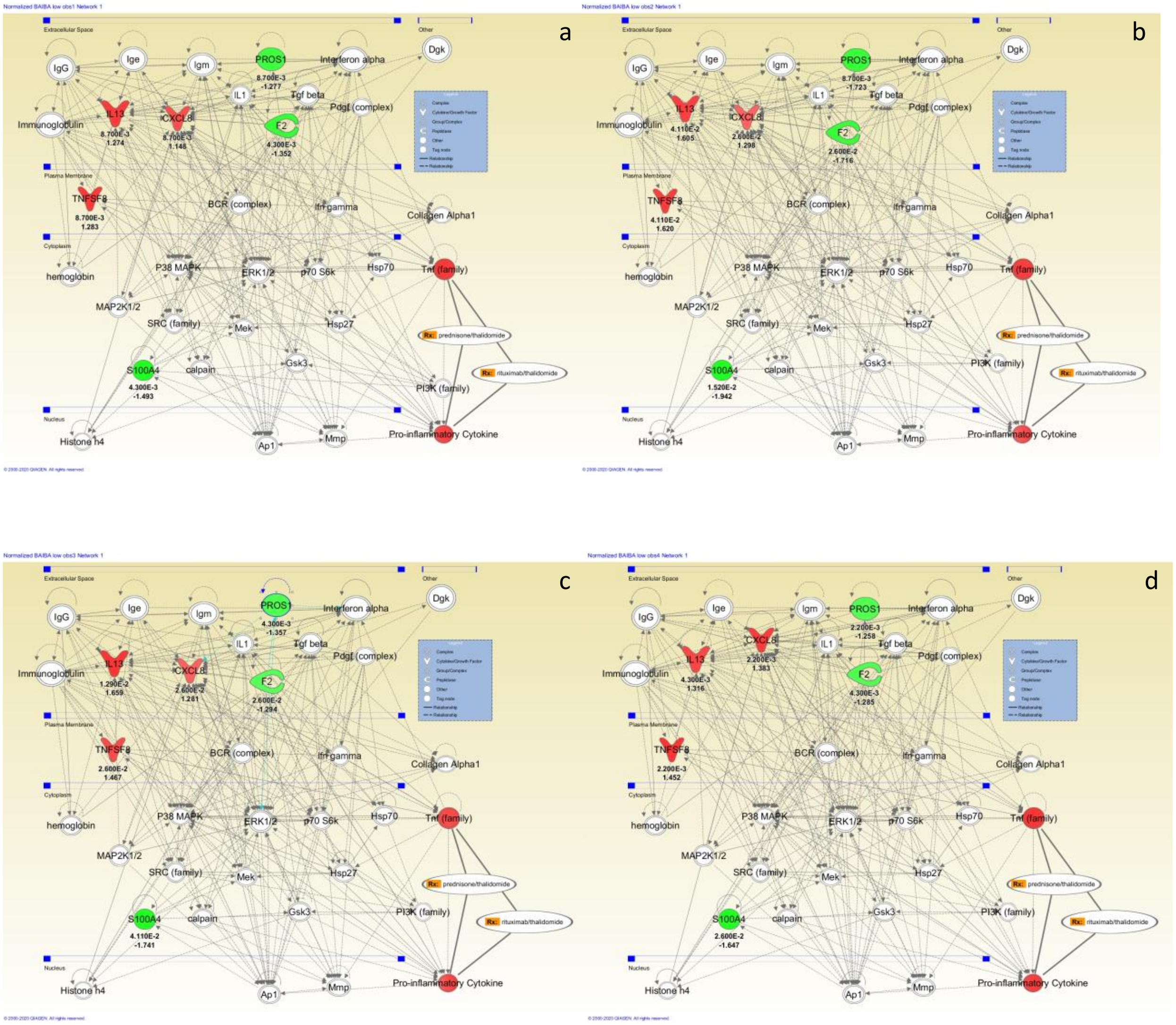

Supplementary Figure 5: IPA Diagrams for BAIBA low dose. Figures: a) BAIBA low dose, b) BAIBA low dose plus prednisolone, c) BAIBA low dose plus rituximab plus prednisolone, d) BAIBA low dose plus rituximab plus vamorolone.

Red indicates upregulation and green indicates downregulation and color intensity correlates with size of expression change. The multicolored elements are protein complexes where the underlying data and documentation are inconsistent with IPAs search algorithms and therefore, the details are not shown.

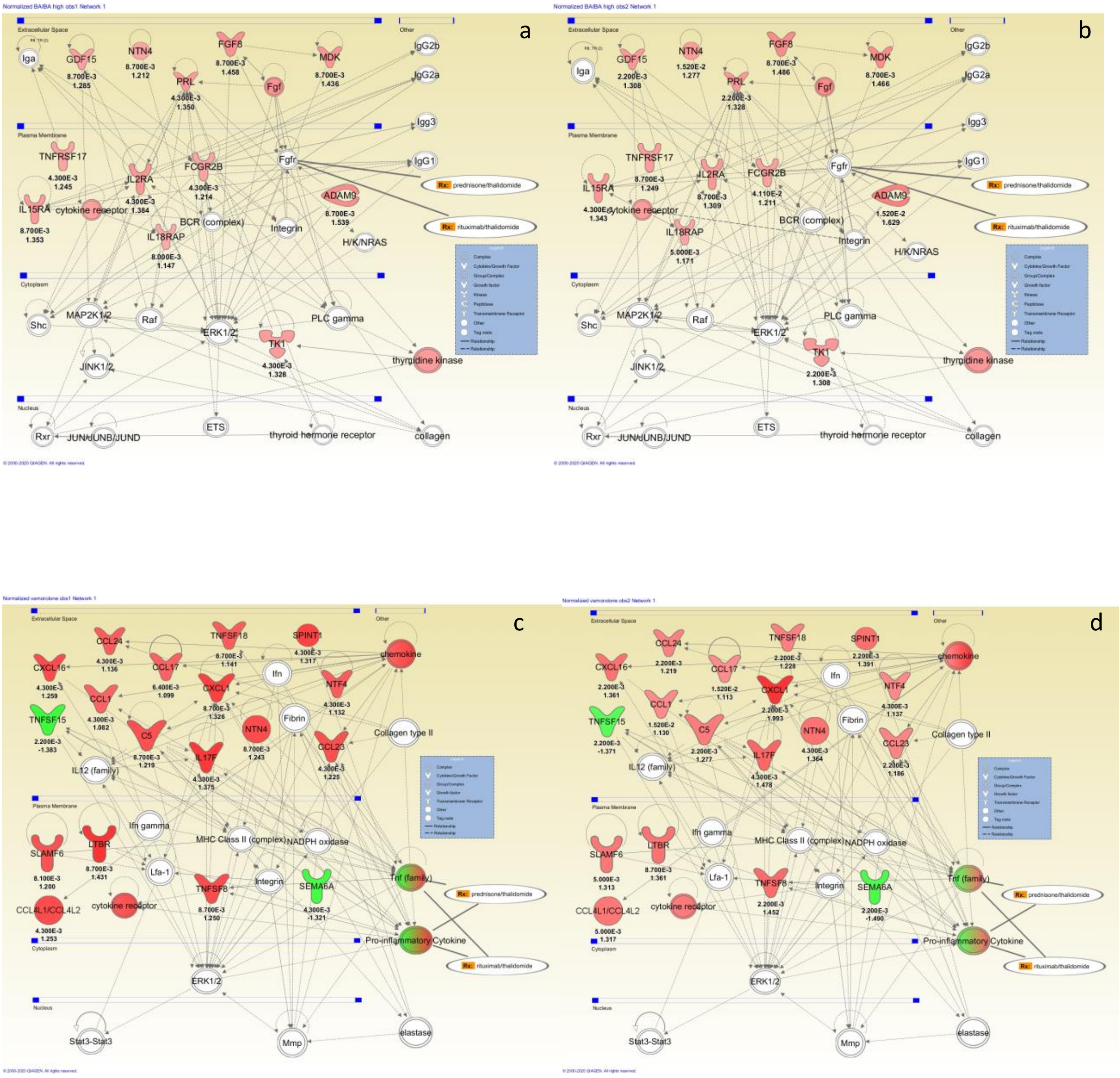

Supplementary Figure 6: IPA Diagrams for BAIBA high dose and Vamorolone. Figures: a) BAIBA high dose, b) BAIBA high dose plus prednisolone, c) Vamorolone, d) Vamorolone plus rituximab plus BAIBA low dose.

Red indicates upregulation and green indicates downregulation and color intensity correlates with size of expression change. The multicolored elements are protein complexes where the underlying data and documentation are inconsistent with IPAs search algorithms and therefore, the details are not shown.
